# Continuous Ammonia Electrosynthesis from Nitrogen and Water in a Monolithic Pd Membrane-Based Flow Cell

**DOI:** 10.1021/acsenergylett.5c03617

**Published:** 2026-01-05

**Authors:** Boxi Ye, Craig Burdis, Vladislav Mints, Yuxiang Zhou, Artem Khobnya, Guanglei Chen, Romain Tort, Johannes Rietbrock, Andreas Kafizas, Mary P. Ryan, Maria Magdalena Titirici, Ifan E. L. Stephens

**Affiliations:** † Department of Materials, 4615 Imperial College London, SW7 2AZ London, United Kingdom; ‡ Department of Chemical Engineering, 4615 Imperial College London, SW7 2AZ London, United Kingdom; § Department of Chemistry, 4615 Imperial College London, SW7 2AZ London, United Kingdom

## Abstract

Continuous electrochemical lithium-mediated ammonia production has shown promising performance. For this reaction, water oxidation could provide a direct route for proton supply, eliminating the need to generate molecular hydrogen. However, recent studies have reported low Faradaic efficiency for ammonia when water is used directly as the proton source. In this work, we integrate an electrically isolated Pd membrane to transfer protons generated from water oxidation into a nonaqueous lithium-mediated nitrogen reduction system. By employing Pd as a proton- and electron-conducting membrane rather than solely as a cathode, we enabled continuous operation in a flow-cell configuration, achieving a Faradaic efficiency of 36 ± 4% at a current density of −6 mA cm^–2^ over 6 h. Online mass spectrometry confirmed that the produced ammonia contained protons generated by water oxidation. This approach to using Pd provides a practical strategy for proton transport and establishes a viable device configuration to advance electrochemical lithium-mediated nitrogen reduction toward sustainable green ammonia synthesis.

## Graphical abstract

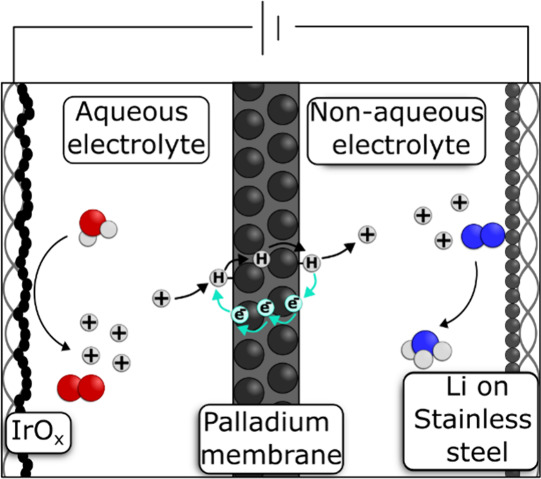

Ammonia (NH_3_), a crucial feedstock for fertilizer production and a potential renewable carbon-free energy carrier, is primarily produced using fossil fuels.^1–3^ Today, 96% of NH_3_ is synthesized via the Haber–Bosch process, which operates at high temperatures and pressures.^4^ Molecular hydrogen (H_2_) is required and is typically derived from natural gas via steam methane reforming, an energy-intensive process.^4^ As a result, the Haber–Bosch process accounts for around 1.3% of global CO_2_ emissions.^5,6^ The high temperatures and pressures required by Haber-Bosch plants necessitate large-scale, capital-intensive facilities concentrated in specific geographical regions.^1^ More than two-thirds of the countries without local Haber-Bosch plants are vulnerable to NH_3_ supply chain shocks.^1,7^ Alternative electrochemical routes to ammonia have consequently been explored as more sustainable and decentralized solutions.^8–11^

Lithium and, more recently, Ca-mediated nitrogen reduction (Li-mediated N_2_ reduction) are the only paradigms irrefutably proven to reduce N_2_ to NH_3_ at a solid electrode.^10,12–16^ Various cell designs have been employed, such as batch,^17,18^ membrane electrode assembly,^19^ and flow cells.^20,21^ In continuous-flow cells, Faradaic efficiencies of up to 76% and operations for hundreds of hours have been demonstrated when anodic H_2_ oxidation supplies protons to the Li-mediated cathode.^20–22^ This outstanding performance has attracted industrial interest in on-site fertilizer production.^17,23^ However, such stability requires a dried and purified H_2_ source.^20,21,24^ Green H_2_ supplied from a water electrolyzer would be ideal for industrial deployment.^22^

Attempts to substitute bottled H_2_ with a water electrolyzer have so far achieved 30% Faradaic efficiency in short-duration tests (8 min) from Lazouski et al.’s work, where residual water contamination at the H_2_ outlet was found to be problematic for NH_3_ production.^22^ Excess water contamination can be a serious issue in Li-mediated N_2_ reduction, inhibiting NH_3_ production.^18,25^

$$2\mathrm{Li}+2H_{2}O\to 2\mathrm{LiOH}+H_{2}$$ 1

The primary problem is that the excessive formation of Lithium hydroxide (LiOH), as described in eq 1.^25^ In our previous paper, we demonstrated that even modest water levels (∼650 ppm) result in the accumulation of insoluble LiOH in tetrahydrofuran (THF), which blocks Li^+^ transport and ultimately suppresses N_2_ reduction over time.^25^ These studies highlight the importance of maintaining a dry, nonaqueous environment. While additional drying of the H_2_ outlet might mitigate this issue, in principle, the entire water electrolyzer could be eliminated if protons generated by water oxidation were delivered directly to the Li-mediated cathode, thereby further simplifying the system and reducing capital costs.^26–28^

Coupling aqueous water oxidation and nonaqueous Li-mediated N_2_ reduction in a single cell (monolithic) is essential for eliminating the need for an external water electrolyzer. Ripepi et al. previously developed a Ni-based electrocatalytic membrane reactor that transported electrochemically generated atomic hydrogen through the metal lattice to hydrogenate adsorbed nitrogen under ambient conditions, achieving a Faradaic efficiency of 0.003% due to limited surface gas phase nitrogen coverage and H_2_ recombination.^26^

Fink et al. demonstrated that a high-surface-area electrodeposited Pd black on a Pd membrane (Pd/Pd black) reactor can couple aqueous water oxidation with catalytic hydrogenation reactions in a nonaqueous environment that enabled electrochemical hydrogenation of anthraquinones to anthrahydroquinones for indirect H_2_O_2_ synthesis at current densities of up to 100 mA cm^–2^, highlighting the potential of Pd membranes to bridge aqueous and nonaqueous chemistries.^29^ Building on this concept, Bemana et al. recently demonstrated the coupling of water oxidation and nonaqueous N_2_ reduction through a hydrogen-permeable Pd membrane via a dual-compartment batch cell. They employed a Pd membrane sandwiched between an aqueous 1 M H_2_SO_4_ electrolyte with an O_2_-evolving Pt counter electrode, and a nonaqueous compartment with 1 M LiBF_4_ in tetrahydrofuran with a 0.5 vol % ethanol electrolyte with a Pt counter electrode, presumably where the organic electrolyte gets oxidized sacrificially.^30^ The Pd served as the cathode on both sides; protons from the aqueous side permeated through the metal lattice to the nonaqueous compartment, where it participated in Li-mediated N_2_ reduction on the Pd surface under ambient conditions, achieving a Faradaic efficiency of 5%.^27,31^ Together, these studies demonstrate the protonation ability of hydrogen-permeable metal membranes when used as cathodes.^27,29,32^ Furthermore, Han et al. showed that Pd can abstract formed during formaldehyde oxidation: the resulting hydride diffuses through the Pd membrane to the opposite side, where it conducts chemical hydrogenation reactions.^33^ However, none of these prior works report a single Pd being used both as anode and cathode simultaneously.

In this work, we demonstrate that by taking advantage of the electronic conductivity of Pd, we can use it as both an anode and a cathode simultaneously, despite being electrically isolated, in a monolithic flow cell for electrochemical N_2_ reduction, enabling continuous conversion of N_2_ and H_2_O to NH_3_ without producing molecular H_2_ as an intermediate (Figure 1). Importantly, we use operando mass spectrometry and isotopic labeling to confirm that the protons in the NH_3_ produced originate from the oxidation of water.

**Figure 1:**
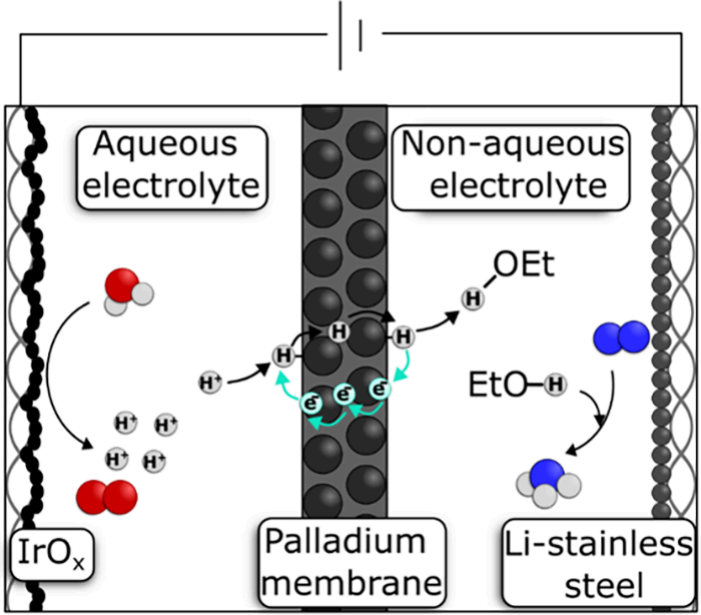
Schematic of Li-mediated N_2_ reduction coupled with water oxidation using a Pd membrane in a monolithic electrolyzer. The aqueous compartment involves the water oxidation at IrO_*x*_ anode, releasing protons that are reduced and adsorbed onto the Pd surface. The adsorbed H atom permeates the Pd lattice and reaches the Li-mediated compartment. The Li-mediated compartment drives the oxidation of atomic H, releasing protons shuttled by EtOH to the cathode for N_2_ reduction.

Li-mediated N_2_ reduction coupled with water oxidation was studied in a custom two-compartment continuous-flow cell (Figure S1). The nonaqueous compartment design was adapted from Fu et al.’s work; the compartment hosted the nonaqueous electrolyte and the gas-diffusion cathode for N_2_ reduction,^21^ while the other compartment contained the aqueous electrolyte to sustain water oxidation. The two compartments were separated by a Pd membrane that selectively transports protons generated during water oxidation while preventing crossover of water, oxygen, and solvated ions. This prevents contamination of the nonaqueous environment, preserving the dry conditions required for Li-mediated N_2_ reduction.^18,25^ A Pd/Pd black membrane, following Fink et al.’s recent work, was used in most of our experiments, as we found it exhibited lower potential losses than bare Pd (see SI for details).^29^

Prehydridation – to preload the membrane with hydrogen atoms – was found to improve Li-mediated N_2_ reduction with Pd membranes (see Figure S2 for the electric circuit connection). Without this step, black precipitates were formed in the nonaqueous electrolyte during the N_2_ reduction reaction, and the cell losses were far more substantial (Figures S3A and S4). The requirement for “pre-hydridation” likely stems from the absence of hydrogen in the Pd lattice at the start and the slow hydrogen permeation kinetics,^34^ leading to increased cell voltage and excessive solvent oxidation (Figure S4). This behavior may reflect findings by Atlan et al., who showed that electrochemically driven hydrogen absorption expands the Pd lattice and alters its phase structure, potentially benefiting membrane performance.^35^ Consequently, unless otherwise stated, all the Pd membranes used in this study were “pre-hydrided”. To maintain a fair comparison with other reported Li-mediated N_2_ reduction systems, we included the charge during the “pre-hydridation” when calculating the Faradaic efficiency toward NH_3_ and energy efficiency for the entire reaction.

To directly verify that the Pd membrane without pretreatment conducts protons, we first performed a controlled proton-transfer experiment in the two-compartment symmetric cell with electrodes geometric surface areas of 2.25 cm^2^ (Figure S2). A two-electrode configuration was used, with IrOx/Ti mesh as the anode and Pt foil as the cathode, separated by a Pd membrane. In the first experiment (Figure 2A), the anolyte contained H_2_O-diluted 0.1 M NaClO_4_, while the catholyte contained D_2_O-diluted 0.1 M NaClO_4_. As charge was passed, the ^1^H NMR signal in the D_2_O chamber increased proportionally with the total charge (30 and 73.2 C), closely matching the theoretical maximum assuming 100% proton transfer (See SI for data processing). This linear relationship confirms that protons generated from water oxidation at the anode successfully permeated through Pd and protonated the D_2_O catholyte. The slightly higher measured H concentration compared to the theoretical maximum is attributed to minor artifacts such as residual protonated species from air exposure, which increase the apparent H content. In the second experiment (Figure 2B), the water distributions were reversed (anolyte = H_2_O; catholyte = D_2_O), and only D^+^ permeates through the membrane. Under these conditions, the ^1^H NMR signal in the D_2_O chamber remained almost constant after 73.2 C of charge was passed, demonstrating negligible H_2_O crossover through Pd. Together, these results provide direct spectroscopic evidence that the Pd membrane selectively transports protons, while effectively suppressing bulk water transfer.

**Figure 2:**
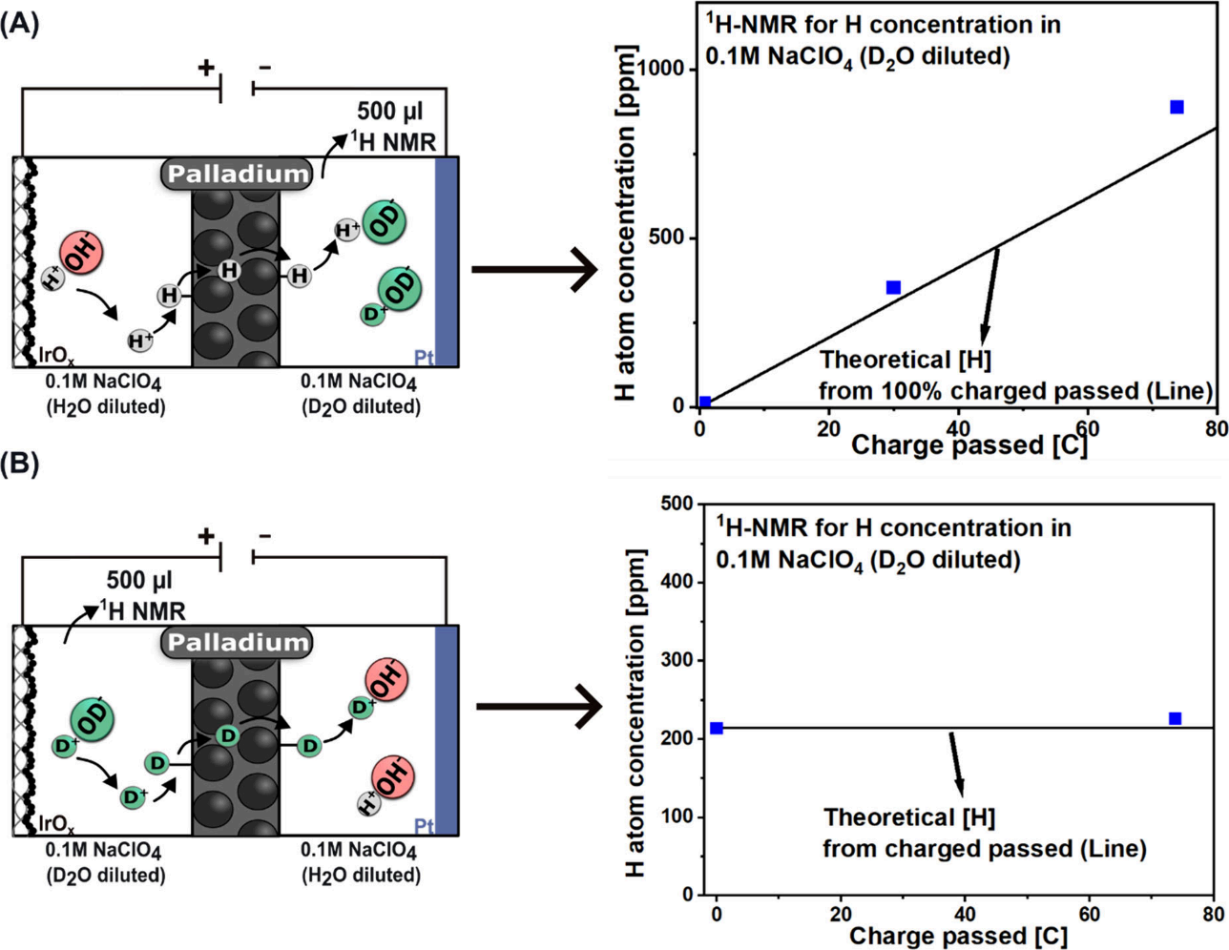
Proton transport through Pd membrane proof of concept. A Pd membrane was used to separate the 0.1 M NaClO_4_ (H_2_O-diluted) chamber and the 0.1 M NaClO_4_ (D_2_O-diluted) chamber. An IrO_*x*_ on Ti mesh and a Pt plate were used as the anode and the cathode, respectively. 500 μL electrolytes in D_2_O were extracted before and after the experiment. (A) Water oxidation performed in the 0.1 M NaClO_4_ (H_2_O-diluted) chamber for 30 and 73.2 C at −9 mA cm^–2^. The H concentration measured (blue squares) aligns well with the theoretical H concentration from 100% charged passed (Line). (B) Water oxidation performed in the 0.1 M NaClO_4_ (D_2_O-diluted) chamber for 73.2 C—negligible H concentration increase after passing 73.2 C charge, indicating no water leakage.

Monolithic N_2_ reduction coupled with water oxidation was carried out first using prehydrided Pd/Pd black as the membrane under the same pulsing current density strategy of −6 mA cm^–2^_geo_ reported by Fu et al. (see Figure 3 and Figure S5A for *iR* corrected cell voltage).^21,29^ For the prehydridation, the Pd/Pd black membrane was connected as the cathode first with only the flow of aqueous electrolyte. Then, 54 C of charge was passed during prehydridation (Figure 2). In our 162 C Li-mediated N_2_ reduction experiment, we produced 270 ± 26 μmol of NH_3_ (Faradaic efficiency = 36 ± 4% (prehydridation charge included) and Faradaic efficiency = 48 ± 5% (without prehydridation charge)) under ambient conditions. The consequence of including the prehydridation charge is that the Faradaic efficiency appears artificially lower at early stages because the fixed 54 C contributes disproportionately to the total charge (See further details in the SI and Figure S6). A conservative upper-bound calculation shows that, even if all EtOH added to the catholyte were fully converted into NH_3_, it could supply at most 210 μmol. What’s more, the ^1^H NMR experiment in Figure 2 proved that the Pd membrane conducts protons. Taken together, these results indicate that ethanol is not the dominant proton donor and that a substantial fraction of the protons incorporated into NH_3_ originates from other proton sources, presumably through the Pd-transported protons.

**Figure 3:**
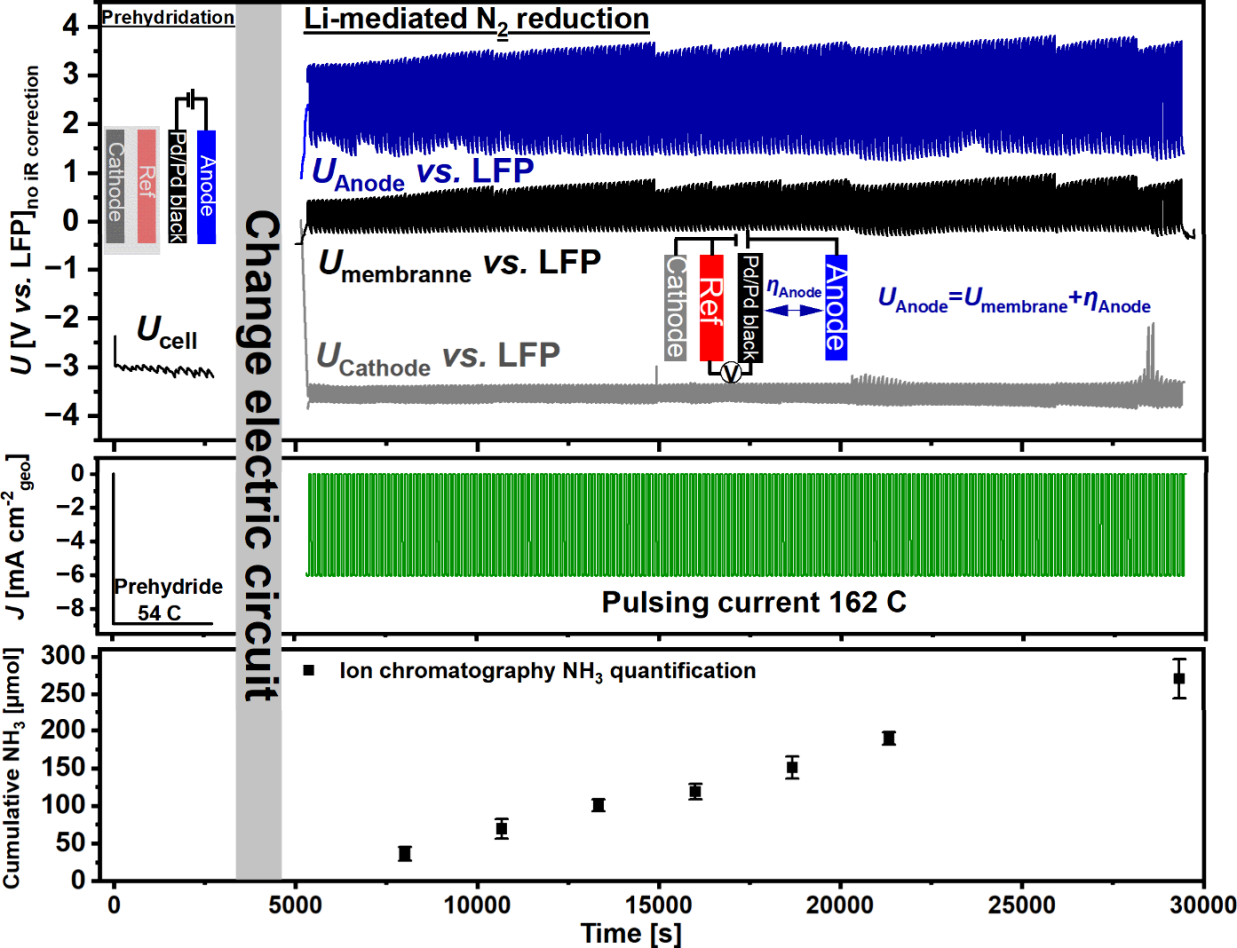
Prehydrided Pd/Pd black membrane operated in a monolithic Li-mediated N_2_ reduction (1 M LiBF_4_ in diglyme with 0.3 vol % ethanol) flow cell coupled with water oxidation (0.1 M NaClO_4_). Top: Electrical configuration and corresponding potentials of *U*_cell_ during prehydridation, *U*_anode_, *U*_membrane_, and *U*_cathode_ vs lithium iron phosphate (LFP) all without *iR* correction during N_2_ reduction. (Note: *U*_anode_ is the sum of *U*_membrane_ and overpotential for anodic reaction (η_anode_)). Middle: applied current densities (prehydridation at −9 mA cm^–2^_geo_ until 54 C had passed; after changing the electric circuit, Li-mediated N_2_ reduction was pulsed at −6 mA cm^–2^_geo_ for 1 min on/1 min off until 162 C had passed). Bottom: cumulative NH_3_ yield measured at various intervals from 18 to 162 C had passed. Error bars denote the standard deviation from *n* = 3 independent experiments.

During N_2_ reduction, stainless steel (cathode), LFP (reference), and IrO_*x*_ (anode) were connected to the potentiostat (Figure 3). Besides, only a voltmeter was connected between the reference and the Pd/Pd black. Therefore, no current was flowing into the Pd/Pd black membrane from the external circuit. During Li-mediated N_2_ reduction, the cell voltage (∼7 V) is attributed to the sum of *U*_cathode_ for Li plating (−3.8 V), *U*_membrane_ for proton transfer (+0.5 V), η_anode_, the overpotential for anodic water oxidation (+1.8 V),^36,37^ and *iR*_drop_ of 1 V. Notably, for the N_2_ reduction electric circuit configuration, the *U*_anode_ measured by the potentiostat would be the sum of the potential drop between the reference and the membrane and the potential difference between the membrane and the anode: *U*_anode_ = *U*_membrane_ + η_anode_. Therefore, from Figure 3, the anode potential (*U*_anode_, +0.4 V from 5000 to 29000 s) rose gradually, which could be attributed to the increase in the membrane potential (*U*_membrane_, +0.4 V from 5000 to 29000 s). The rise in *U*_membrane_ may reflect the gradual deactivation of Pd/Pd black surface sites, potentially due to (i) a limited proton supply from the unoptimized aqueous 0.1 M NaClO_4_ compartment, or depletion of adsorbed H atom into H_2_ gas;^29^ (ii) adsorption of organic species in the nonaqueous compartment, blocking sites for H adsorption;^38^ or (iii) Pd surface dissolution.^39^ Our benchtop XRD did not verify the peak shift of the membrane after the experiment (see Figure S7). Therefore, these degradation mechanisms will be investigated further in future work by using other characterization techniques.^40–42^

The *U*_membrane_ reached a maximum of +0.9 V vs lithium iron phosphate (LFP), below the potential at which Mygind et al. reported the onset of diglyme oxidation, +1 V vs LFP.^43^ However, the nonaqueous electrolyte became cloudy after 29000 s (Figure S3B). We hypothesize that the cloudiness arose from the formation of a thick solid–electrolyte interphase (SEI) (Figure S3C), flushed into the flowing electrolyte by our unoptimized pulsing strategy. We conjecture that imposing longer duration rest potentials should prevent the buildup of excessive Li and SEI layers.^20,44^

Solvent oxidation may also occur under reaction conditions. However, we did not observe any distinct peaks in ^1^H NMR. Nonetheless, we acknowledge that most oxidation products could overlap with the chemical shift of diglyme (see Figure S8).^45^ Therefore, complementary studies using mass spectrometry and various membranes were conducted to elucidate the mechanism further and confirm the proton source. Furthermore, we predict that the membrane potential would continue to ramp up with longer-duration measurements (more than 162 C), leading to eventual solvent or membrane degradation. Therefore, to protect the Pd membrane, each experiment was limited to the duration required for the membrane potential to reach +1 V vs LFP. Before every Li-mediated N_2_-reduction experiment, the Pd membrane was first hydrided to ensure a well-defined initial state, and after each experiment, it was cleaned with 1 M HNO_3_ to remove surface impurities.^29^

To investigate the proton source for the Li-mediated N_2_ reduction reaction, we connected the flow cell to a commercial, chip-based online mass spectrometer (see the SI for detailed settings and Figure S9).^46^ We examined the gas products of the Li-mediated N_2_ reduction, which primarily consist of NH_3_ and H_2_ (see all other measured signals in Figure S10).^47^ In this set of experiments, the Pd/Pd black membrane was compared with a Pt membrane (both prehydrided). All the MS signals were exported as raw data without any calibration. Therefore, we have only performed a qualitative analysis of the trends. A moderate current density of (−2.7 mA cm^–2^) was used for mass spectrometry experiments to allow a significant signal to rise while avoiding extreme solvent degradation with a Pt membrane. Krempl et al. had found that solvent degradation could lead to electrolyte acidification and trap more gas-phase ammonia as ammonium, which may affect signal intensity in our mass spectrometer.^48^

For Pd/Pd black, when H_2_O was being used as a solvent in the anode chamber (Figure 4A), an increase for the *m*/*z* = 2 and *m*/*z* = 17 signals confirmed the detection of H_2_ and NH_3_, respectively. For H_2_ (*m*/*z* = 2), the signal first spiked during the initial LSV, likely owing to the reduction of ethanol, which is reported to happen 1.5 V positive of lithium plating.^49^ The *m*/*z* = 2 signal fluctuation was due to manual adjustments to the N_2_ gas flow rate, which was sometimes caused by flooding of the gas diffusion electrode, affecting the detected gas concentration. The signal for NH_3_ (*m*/*z* = 17) was slightly delayed due to the formation of the solid electrolyte interphase and the tendency of NH_3_ to adsorb onto the MS inlet tubing.^50^ While all the measured signals began to drop when the current was switched off after 1800 s, the signal for NH_3_ (*m*/*z* = 17) continued to increase without any external current supply until 2700 s. This observation aligns well with the findings reported by Krempl et al., who observed a similar trend and proposed that the continued formation of NH_3_ could result from either N_2_ reduction mediated by electronically isolated ’dead lithium’ or from the chemical decomposition of accumulated LiN_*x*_H_*y*_ intermediate species on the electrode surface.^47^

**Figure 4:**
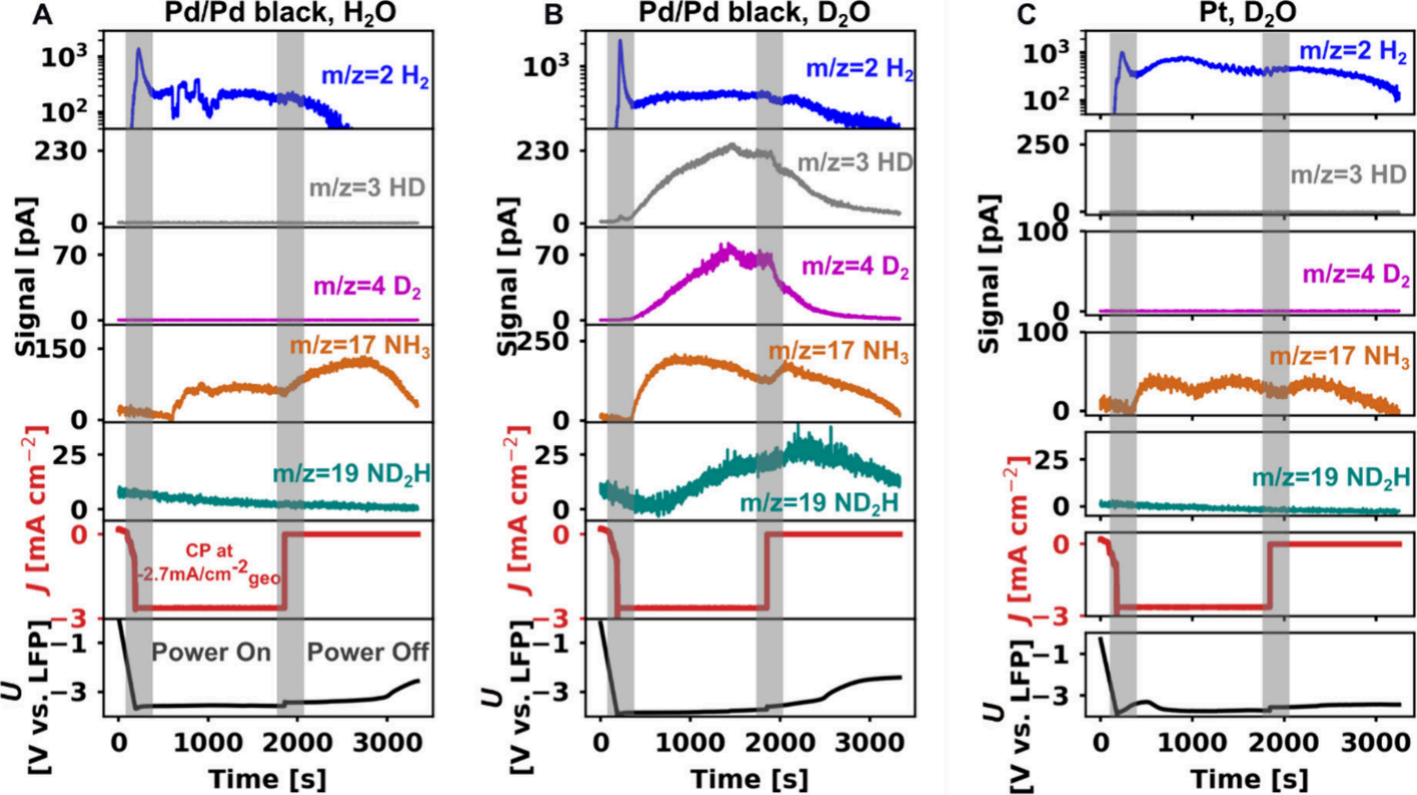
Online mass spectrometry of NH_3_, H_2_, and the relevant deuterated species for Li-mediated N_2_ reduction coupled with water oxidation. The top five panels display the MS current signals corresponding to NH_3_ species (*m*/*z* = 17 and 19) and hydrogen species (*m*/*z* = 2, 3, and 4). The H_2_ signal was plotted on a logarithmic scale to accommodate its wide range and reveal clearer trends. The bottom two panels show the corresponding current density and potential during LSV scanning the cathode potential from 0 V to −4 V vs LFP at a scan rate of 20 mV s^–1^, terminating when the current density (*J*) reached −2.7 mA cm^–2^_geo_. Then, a constant current density of −2.7 mA cm^–2^_geo_ was applied for 1667 s (total charge = 10 C), followed by 30 min at open-circuit. The shaded area represents the moment when the current supply was turned on and off. (A) A Pd/Pd black membrane with H_2_O as proton source. (B) A Pd/Pd black membrane with D_2_O as the deuteron source. (C) A Pt membrane with D_2_O as the deuteron source.

To probe the source of the protons in the produced NH_3_ for the Pd/Pd black membrane, H_2_O was replaced with D_2_O. Signals for deuterated NH_3_ and H_2_ indicate that protons from anodic water oxidation were transported and utilized in the cathode compartment, as shown in Figure 4B. Initially, H_2_ (*m*/*z* = 2) and NH_3_ (*m*/*z* = 17) were detected. As more charge passed, the signals for *m*/*z* = 3 and *m*/*z* = 4 increased, indicating the evolution of HD and D_2_. Initially, NH_3_ was produced with protons, rather than deuterons, because the nonaqueous catholyte (1 M LiBF_4_ in diglyme with 0.3 vol % ethanol) contained a proton source from the ethanol. As the reaction proceeded, deuterium was transported through the Pd membrane, enabling the formation of HD, D_2_, and ND_2_H. The delayed appearance of the deuterated species could be attributed to the time required for the depletion of the electrolyte’s initial H content and replenishment with D.^21^ The same trend of increased ND_2_H (*m*/*z* = 19) after current termination further suggests that the species was likely to be deuterated NH_3_. We could also expect to detect other deuterated NH_3_ species, such as NDH_2_ and ND_3_ (*m*/*z* = 18 and 20). However, our system could not distinguish them. NDH_2_ (*m*/*z* = 18) overlaps with the H_2_O signal, which also has a *m*/*z* = 18. Since our MS was not in an inert atmosphere, it inevitably collected background moisture. We expect that extending the electrolysis duration could lead to the emergence of an ND_3_ signal (*m*/*z* = 20), as the current system likely retains a significant number of residual protons, which limits the formation of fully deuterated NH_3_.

For comparison, the Pt membrane was also tested using D_2_O as a deuteron source, as shown in Figure 4C. It was confirmed that only H_2_ (*m*/*z* = 2) and NH_3_ (*m*/*z* = 17) were produced, indicating that negligible deuterons were transported through the Pt membrane. The signals further confirm that all the NH_3_ produced originates from the proton source in the nonaqueous compartment, rather than from water.

Different membranes exhibit distinct behaviors. We tested bare Pd and Pd/Pd black to investigate how hydrogen availability on the membrane surface in the nonaqueous compartment influences the membrane potential. For further comparison, prehydrided Pt and bare Nafion were included due to Pt’s negligible atomic hydrogen permeability and Nafion’s water permeability, enabling us to study distinct reaction mechanisms for each membrane.^51,52^ For consistent comparison, all the membranes were tested under identical electrochemical conditions, as shown in the bottom panel of Figure 5A. The anode, membrane, and cathode potentials vs LFP were continuously monitored throughout both the LSV and the subsequent constant current step, during which 10 C of charge was passed for NH_3_ production. The current density (−1.75 mA cm^–2^_geo_) was selected to minimize the side effect of solvent oxidation.^43^ Notably, differences in anode potential among the membranes mirror the differences in membrane potentials, indicating stable IrO_*x*_ anode performance (see Figure S5B for different membranes’ cell voltages).

**Figure 5:**
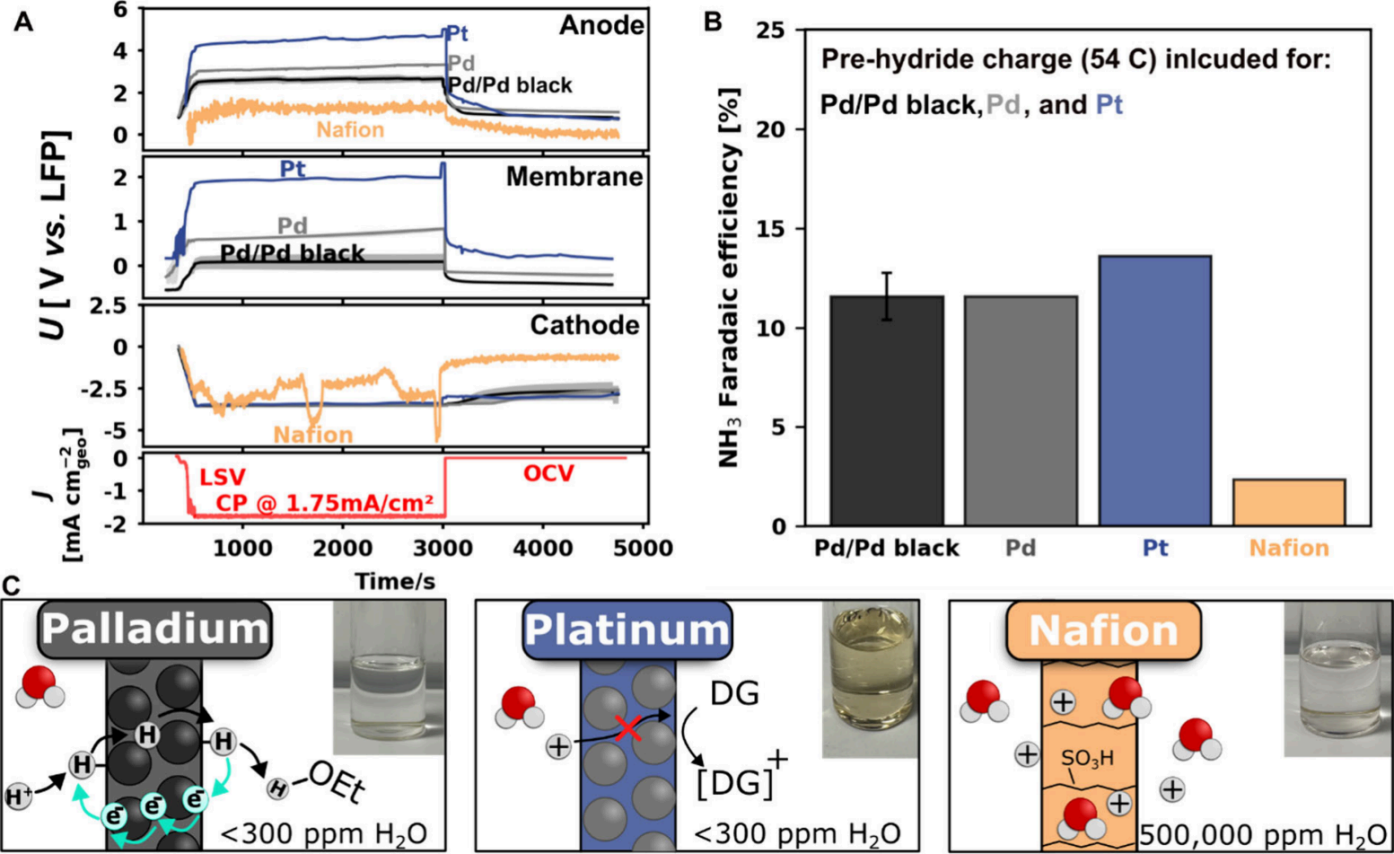
Electrochemical performance of Li-mediated N_2_ reduction coupled with water oxidation using different membranes. (A) 1st panel: Anode potential profiles. 2nd panel: Membrane potential profile. 3rd panel: Cathode potential profile. 4th panel: Current density profile. The method: an initial LSV from 0 V to −4 V vs a LFP reference at a scan rate of 20 mV s^–1^, followed by a constant current density of −1.75 mA cm^–2^_geo_ for 2500 s (total charge = 10 C) and then open-circuit hold. The black, gray, blue, and orange curves represent Pd membrane electrodeposited with Pd black (Pd/Pd black), a bare Pd membrane, a Pt membrane, and a Nafion membrane, respectively. Shaded areas indicate the standard deviation of the independent measurements. (B) NH_3_ Faradaic efficiency of each membrane configuration under identical electrolysis conditions (−1.75 mA cm^–2^_geo_, 2500 s). Error bars denote the standard deviation from *n* = 3 independent experiments. (C) Images of the electrolytes’ color after 10 C with the proposed reaction mechanisms.

The Nafion membrane potential could not be measured as it is not electrically conducting. For the cathode potential, the other membranes exhibited a stable potential consistent with lithium plating (at approximately −3.4 V vs LFP),^53^ whereas the Nafion case showed a highly noisy profile and failed to maintain this potential, Figure 5A. This behavior is likely caused by the extremely high water content in the electrolyte (∼400000 ppm after the experiment), which promotes side reactions and prevents lithium plating (Figure 5C). As expected, negligible NH_3_ was detected when using Nafion, with a Faradaic efficiency of 1.2% (Figure 5B).^18,25^

At a constant current, Pt operates at a significantly higher membrane potential and remains stable at approximately +2 V vs LFP (considerably higher than the 1 V vs LFP attributed to diglyme oxidation reported by Mygind et al.)^43^ throughout the electrolysis, Figure 5A. This suggests that the Pt membrane is oxidizing the electrolyte throughout the whole experiment, even though the Faradaic efficiency toward NH_3_ was 15% (Shown in Figure 5B).^21,22,31^ The yellow color of the electrolyte observed after passing 10 C of charge (Figure 5C) provides visual evidence of solvent degradation. Although the color of degraded diglyme is not well reported,^45^ a similar phenomenon was observed by Fu et al., who reported substantial solvent degradation during their flow cell experiments in the absence of hydrogen, attributing it to THF decomposition at an anode potential of +1.7 V vs Pt.^21^ Likewise, Du et al. showed that under high-pressure N_2_ (15 bar), anodic oxidation of THF produces reactive intermediates that irreversibly react with alcohol proton carriers.^31^

The Pd/Pd black exhibited a lower membrane potential (0 V vs LFP) than the bare Pd membrane (+0.6 V vs LFP), due to its increased surface area, which provides more active sites for hydrogen adsorption.^29^ Furthermore, the visually unchanged electrolyte further suggested that the reaction on both Pd membranes involved proton release rather than electrolyte oxidation. However, because approximately 65% of the charge for these experiments was directed toward the initial “pre-hydridation” process, all the membranes exhibited a low Faradaic efficiency of less than 20% (see SI for the effect of prehydridation charge on Faradaic efficiency over time and SI, Table 2 for summarized Faradaic efficiency without prehydridation charge).

Comparison among reported studies. Figure 6 compares all Li-mediated and membrane-mediated N_2_ reduction systems discussed previously in terms of energy efficiency (%), Faradaic efficiency (%), duration (hours), and average current density (*J*_average_, mA cm^–2^_geo_) (accounting for different pulsing strategies applied).^20,22,27^ So far, Li-mediated N_2_ reduction systems supplied with pure H_2_ have demonstrated the best overall performance, achieving high Faradaic efficiency, energy efficiency, and stability.^20^ In contrast, membrane-based ammonia synthesis, in which the proton source is water directly, has only recently emerged as a new direction and remains at an early stage of development.^27^ Nevertheless, by eliminating water crossover, our system achieves greater stability than a water electrolyzer connected in series with the Li-mediated N_2_ reduction cell.^22^ Employing Pd as a cathode in the batch cell enabled a monolithic device for combined water oxidation and N_2_ reduction.^27^ We found that Pd can also function as an anode, enabling continuous operation in our monolithic flow cell and achieving a Faradaic efficiency of 36 ± 4%. With further optimization of the membrane and electrolyte, we foresee that this system could match or even surpass the performance of the conventional pure H_2_-fed Li-mediated setup.^20^

**Figure 6:**
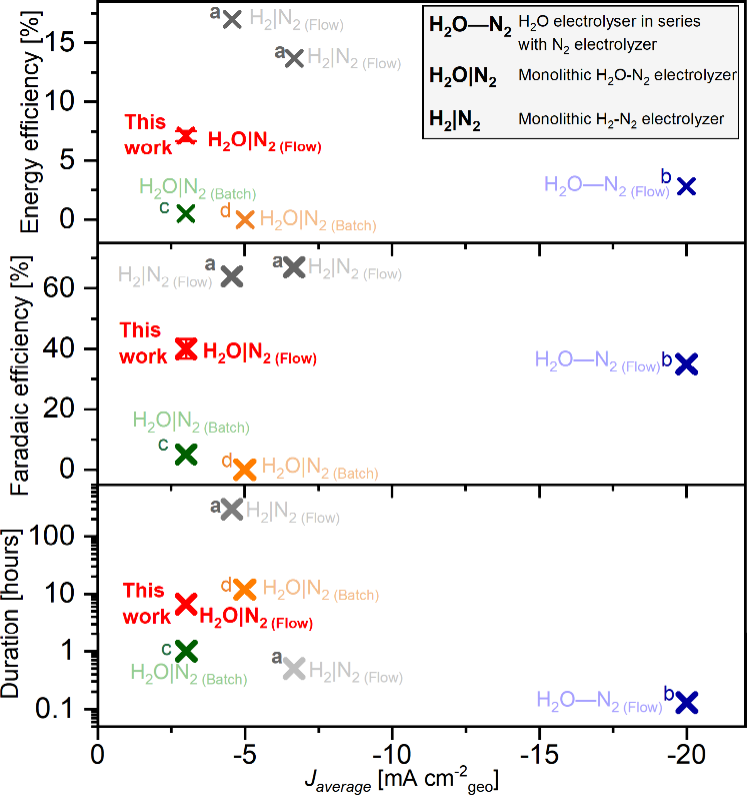
Energy efficiencies (%), Faradaic efficiencies (%), Duration (hours) of Li-mediated N_2_ reduction against average current densities (*J*_average_, mA cm^–2^_geo_) in reported literature: this work: monolithic H_2_O–N_2_ flow electrolyzer (red), a: monolithic H_2_–N_2_ flow electrolyzer (gray and light gray),^20^ b: H_2_O electrolyzer in series with N_2_ electrolyzer (blue),^22^ c: H_2_O–N_2_ batch electrolyzer (green),^27^ and d: H_2_O–N_2_ batch electrolyzer (orange).^26^

This work establishes an electrically isolated hydrogen-permeable membrane (Pd) in facilitating NH_3_ production in a flow-cell device under ambient conditions. The observed requirement for membrane prehydridation and the gradual increase in membrane potential during pulsed operation indicate that hydrogen-transfer kinetics and membrane stability are key factors limiting performance. In our system, the neutral aqueous electrolyte is optimized for D_2_O isotopic labeling rather than to minimize potential losses; however, a more acidic medium, such as 1 M H_2_SO_4_, which has shown nearly complete proton permeation across Pd membranes in related systems,^29^ will yield more energy-efficient operation. Further improvements could involve employing alternative hydrogen-permeable metals and alloys, which may enhance permeability and durability and reduce costs.^54–56^ In addition, advanced characterization techniques, such as synchrotron X-ray diffraction (synchrotron XRD),^40^ scanning electron microscopy (SEM),^41^ and atom probe tomography (APT),^42^ could be used to elucidate structural evolution, hydrogen distribution, and membrane degradation pathways in greater detail. The membrane configuration we report here will likely have applications beyond NH_3_ synthesis, in other electrochemical transformations that require anhydrous environments yet rely on controlled proton delivery, such as CO_2_ reduction and nonaqueous redox-flow batteries.^57,58^

## Supplementary Material



## Notes

Materials, experimental methods, electrochemical cell setup and measurement information, NH_3_ quantification data, and tabulated values for raw experimental data (PDF)
