# Supplementary material for: Continuous Ammonia Electrosynthesis from Nitrogen and Water in a Monolithic Pd Membrane-Based Flow Cell
Source: ACS Energy Lett. 2026 Jan 5;11(2):1907–15. doi: 10.1021/acsenergylett.5c03617 (PMC12910716; doi:10.1021/acsenergylett.5c03617)
Supplement: Supplementary file 1 [file nz5c03617_si_001.pdf]

Supplementary Materials for

Continuous ammonia electrosynthesis from nitrogen and water in a monolithic Pd membrane-based flow cell

*Boxi Ye,[a] Craig Burdis,[a] Vladislav Mints,[b] Yuxiang Zhou,[a] Artem Khobnya,[a] Guanglei Chen,[a] Romain Tort,[a] Johannes Rietbrock,[a] Andreas Kafizas,[c] Mary Ryan,[b] Maria Magdalena Titirici,[b] and Ifan Stephens[a]**

[a] Department of Materials, Imperial College London, SW7 2AZ London, UK

[b] Department of Chemical Engineering, Imperial College London, SW7 2AZ London, UK

[c] Department of Chemistry, Imperial College London, SW7 2AZ London, UK

*Corresponding author. Email: i.stephens@imperial.ac.uk;

Table of Contents

1. Materials and methods **3**
1.1 Materials 3
1.2 Electrolyte Preparation 3
1.3 Electrodeposited Pd black on Pd foil (Pd/Pd black) preparation 3
1.4 Anode spray-coated IrO_x preparation 3
1.5 Reference lithium iron phosphate (LFP) preparation 4
1.6 Ammonia (NH_3) quantification 4
1.7 Water content detection 4
1.8 ^{1}H NMR for H concentration in D_2O 5
1.9 Physical Characterization (XRD) 7
2. Flow cell experiments **7**
2.1 54C prehydride+162C N_2 reduction flow cell experiments: **8**
2.2 10C N_2 reduction flow cell experiments: **8**
2.3 Blank experiment for water concentration: **8**
3. Flow cell connected to an electrochemistry mass spectrometry experiment (EC-MS) **8**
4. Calculations **9**
4.1 Faradaic efficiency (FE) 9
4.2 Energy efficiency 10
4.3 Average current density 10
4.4 Effect of including pre-hydridation charge of 54 C during Faradaic efficiency (FE) calculation 10
5. Figures **12**
6. Tables **22**
References **23**

1. Materials and methods

1.1 Materials

Palladium foil (99.99%) with a thickness of 0.025 mm was purchased from Advent Research Materials and used as a proton-selective membrane. Pt membrane and back plate (Pt foil 99.95 %, 25 µm thick, Goodfellow Cambridge). Nafion 211 membrane (50 µm, FuelCellStore). Stainless steel mesh (cathode, McMaster Carr: 325* 2300 mesh, 5µm pore size, 70 µm thick). Anode: IrOx (Iridium (IV) oxide, 99.9% trace metals basis, Sigma Aldrich). Platinized Ti Fiber Felt (Fuel Cell Store, Porosity=53-56%), and $LiFePO_4$ coated on Al sheets (BR0188, 135 mA h g^{-1} capacity, 28 mg cm^{-2} loading) purchased from MSE supplies were cut to 18 mm diameter discs for usage. $NaClO_4$, (Sigma-Aldrich, Sodium perchlorate hydrate, 99.99% trace metals basis). $PdCl_2$, (Sigma-Aldrich, Palladium (II) chloride ⩾99.9%). Rubidium nitrate (Sigma Aldrich; RbNO3: 99.7 % trace metal basis), Sodium carbonate (Sigma Aldrich; Na2CO3: anhydrous), Sodium hydrogen carbonate (Sigma Aldrich; NaHCO3). Hydrochloric acid (Sigma Aldrich; HCl: 30 %), Nitric acid (Sigma Aldrich; HNO3: 70 %, ACS reagent). Isopropanol (2-Propanol ≥99.7%, VWR Chemicals). Nafion ionomer (Perfluorinated resin solution, 5wt% in lower aliphatic alcohols and water, contains 15-20% water, Sigma Aldrich). D_2O (Sigma Aldrich, Deuterium oxide, 99.9%), Ultrapure water (Sartorius, 18.2 MΩ resistivity).

Items stored in Ar glovebox: Diglyme (Sigma Aldrich; 2-Methoxyethyl ether: anhydrous, 99.5 %), Ethanol (Acros Organics; EtOH: extra dry, absolute, 99.5 %), Lithium tetrafluoroborate (Stanford Advanced Materials; $LiBF_4$: ≥99 %).

Ar (BOC, 99.9999%) and N_2 (BOC, 99.9999%) were purified using NuPure purifiers, which reduced contaminant levels of H_2O, H_2, CO_2, O_2, CO, and non-methane hydrocarbons. (NMHC), CH4, NH3, NOx to < 0.5 ppb.

1.2 Electrolyte Preparation

$LiBF_4$ was dried for 72 hours at 80 °C under vacuum (-0.1 mbar) to remove residual water and other contaminants. Non-aqueous electrolytes (1M LiBF4 in diglyme with 0.3 vol% Ethanol) were prepared in an argon-filled glovebox (MBraun, H2O < 0.3 ppm, O2 < 0.3 ppm). Aqueous electrolytes (0.1M $NaClO_4$) were prepared in a fume hood.

1.3 Electrodeposited Pd black on Pd foil (Pd/Pd black) preparation

Pd/Pd black was prepared as reported by Fink et al. [1], but in a flow cell:
The Aqueous compartment was filled with 8 ml of 15.9 mM $PdCl_2$ in 1 M HCl. A two-electrode setup was used: Pd (Cathode) and spray-coated IrO_x (Anode, reported in Section 1.4). Then, -9 mA $cm^{-2}{}_{geo}$ constant current was applied to the cathode until 30 C of charge had been passed. Then repeat the constant current step one more time. Therefore, a total of 60 C of charge passed. After every experiment, the Pd membranes were cleaned using 1 M HNO_3. None of the Pd/Pd black samples was used for more than 10 experiments. However, we advise that gentle mechanical abrasion followed by redeposition will be required if Pd/Pd black is used for more than 10 experiments [1].

1.4 Anode spray-coated IrO_x preparation

Spray-coated IrO_x on platanized Ti fibre felt was prepared as reported by Ismail et al. [2]:

IrO_x particles were dispersed in a 1:1 (v/v) mixture of water and isopropanol at a concentration of 20 mg ml⁻¹, with Nafion ionomer (5 wt%) added at 15 m/m% % relative to the combined mass of catalyst and ionomer. The suspension was sonicated for 25 minutes in an ultrasonic bath maintained at a temperature below 20 °C. The resulting dispersion was spray-coated onto a preheated titanium screen at 85 °C using a handheld airbrush. The anode catalyst loading was 1.2 ± 0.1 mg cm⁻².

1.5 Reference lithium iron phosphate (LFP) preparation

LFP reference was prepared as reported by Tort et al. [3]:
In an Ar-filled glovebox, a Li disc was mounted on a stainless-steel spacer and spring in the negative case of a coin cell. An 18 mm diameter disc was cut from the $LiFePO_4$ sheet, placed in the positive case, and covered with a separator wetted with 70–100 µl of electrolyte (1 M $LiBF_4$ in diglyme). The cell was sealed using a 7-bar press. The $LiFePO_4$ coin cells were discharged at 1.56 mA g^{-1} (0.01C) to a cut-off voltage of 4.0 V vs. Li. The cells were then allowed to relax to a potential plateau of +3.428 ± 0.003 V *vs.* Li, which remained stable for several days in the coin cell.

1.6 Ammonia (NH_3) quantification

Both NH_3 trapped in the non-aqueous electrolyte and as ammonium ions NH_4^+ in the acid trap were quantified by ion chromatography following the method of Burdis et al. [4]:
A Metrohm Eco IC, equipped with an autosampler and a Metrosep C6 column, was used. The eluent consisted of 5.0 mM nitric acid and 0.34 µM rubidium nitrate, while the regenerant was 70 mM sodium carbonate and 70 mM sodium bicarbonate. Samples were diluted 10–100 fold to ensure concentrations fell within the calibration range and to extend the lifetime of the column and instrument.

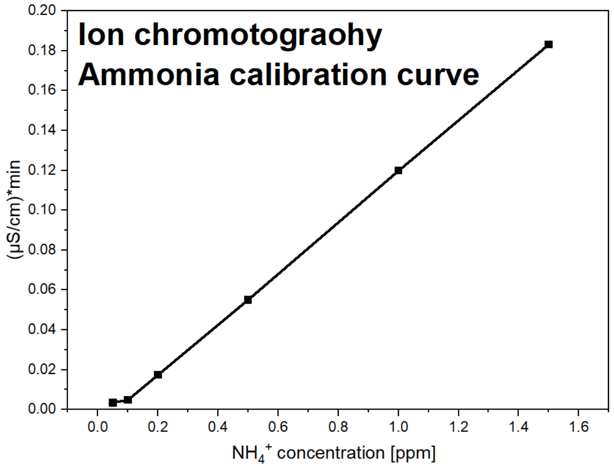

1.7 Water content detection

A Karl Fisher titrator (C20, Mettler Toledo) was used to determine the water concentration for all non-aqueous electrolytes after flow cell experiments. For samples collected after tests with a Nafion membrane, the non-aqueous electrolyte was diluted with dried diglyme to protect the instrument and keep readings within range. We did this by diluting samples after Nafion membrane experiments, 400, 800, and 1600 times with dried diglyme, respectively. The actual water content was calculated by first converting the values from the 400×, 800×, and 1600×

diluted samples back to their original (undiluted) concentrations, and then averaging the results (See Table 1).

1.8 ^{1}H NMR for H concentration in D_2O

Calibration sample preparation. To establish a calibration curve for ^{1}H concentration in D_2O, a series of D_2O–$NaClO_4$ electrolytes with known H_2O contents was prepared. All solutions contained 0.1 M $NaClO_4$ in 99.9% D_2O (nominal H_2O content 1000 ppm). Additional volumetric additions of H_2O were made to obtain standards of 3000, 5000, 7000, 9000, and 11000 ppm. Each standard (500 µL) was transferred into a 5 mm NMR tube and sealed with a PTFE-lined cap to minimize atmospheric moisture uptake.
Instrument and acquisition settings. ^{1}H-NMR spectra were recorded on a Bruker AV-400 spectrometer (400 MHz, 25 °C) using a standard 5 mm broadband probe. To ensure comparable quantitative conditions across all samples, each spectrum was collected using a single scan (NS = 1) with a 90° pulse, a relaxation delay of 10 s, and an acquisition time of ~4 s.
Data processing. Spectra were processed using MestReNova software.

Calibration curve for "Moles of H atoms vs. ^{1}H NMR area under the peak".
The integral of the peak area between (δ=4.6 to 4.8) was taken as "Area under the curve (absolute)" for H content quantification. For example, Sample A:

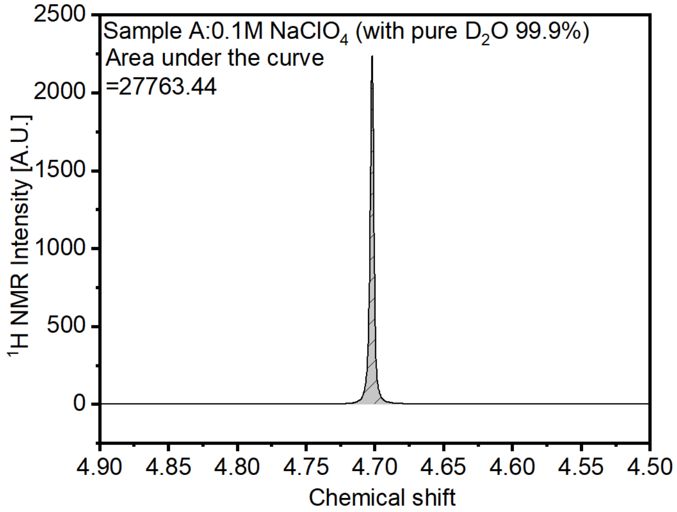

We know the H_2O concentration of the prepared samples. We can convert the H_2O concentration into the concentration of H atoms by using the formula:

$$[H]_{ppm} = \left(\frac{2}{18}\right) * [H_2O]_{ppm}$$

See the calibration sample list below:

Sample (500 µL)	H_2O concentration standard solution (ppm)	Converted H atom concentration [H] (ppm)	Absolute area under the curve
A	1000	111.1	27763.44
B	3000	333.3	67674.47
C	5000	555.6	109928.06
D	7000	777.8	148241.38
E	9000	1000	186158.23

F	11000	1222.2	221126.63

A calibration curve for “Absolute area under the curve *vs*. H atom concentration” can then be plotted as:

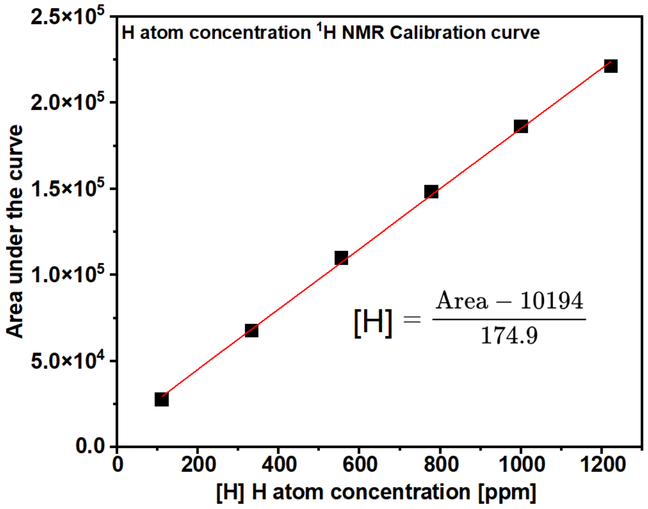

Electrochemistry measurement for NMR quantification

A two-compartment cell, schematically shown in Figure S1, was used for the proton-transport measurements. In contrast to the flow-operated configuration, the electrolyte was manually introduced into each compartment using a syringe; therefore, the cell was operated in batch mode. A palladium foil separated the two chambers, and all ports, except one on each side, were sealed with screw fittings to prevent electrolyte leakage during filling.

Approximately 1 ml of 0.1 M $NaClO_4$ prepared in H_2O was injected into one compartment, while another 1 ml of 0.1 M $NaClO_4$ prepared in D_2O was introduced into the opposite compartment. In the first experiment, the H_2O-containing chamber was connected as the anode, and a constant current of -20 mA (-9 mA cm^{-2}) was applied for 25 min. The cell was then fully disassembled, cleaned, reassembled, and refilled with fresh electrolytes. A second galvanostatic experiment was performed under identical conditions except that the electrolysis time was extended to 1 h at -20 mA (-9 mA cm^{-2}).

After completing these measurements, the electrode connection was reversed: the D_2O-containing compartment was connected as the anode, while the H_2O-containing chamber served as the cathode. This reverse-bias experiment was conducted at a current of -20 mA (-9 mA cm^{-2}) for 1 hour and under the same electrolyte conditions to assess whether water is leaking into the Pd membrane.

For all experiments, 500 µL of electrolyte was collected from the D_2O compartment before and after electrolysis for ^{1}H NMR analysis to quantify the transferred proton content.

The following table summarizes the raw electrochemical and NMR data:

Anode chamber electrolyte	Cathode chamber electrolyte	Charge passed [C]	NMR area before	NMR area after	$\Delta[H]$ [ppm]
0.1 M $NaClO_4$ (H_2O)	0.1 M $NaClO_4$ (D_2O)	30	375489	437475	354

0.1 M $NaClO_4$ (H_2O)	0.1 M $NaClO_4$ (D_2O)	73.2	51321	207000	890
0.1 M $NaClO_4$ (D_2O)	0.1 M $NaClO_4$ (H_2O)	73.2	47682	49740	12

The theoretical line of H atom concentration [H] from 100% charge passed in 1ml (0.001 L) of electrolyte is calculated as:

$$[H]_{ppm} = \frac{Q * Mr_H * 1000}{F * 0.001}$$

Where Q is the total charge passed during water oxidation [C]; Mr_H is the molar mass of hydrogen g mol^{-1}; F is Faraday's constant=96485 [C mol^{-1}].

1.9 Physical Characterization (XRD)

XRD measurements were performed on a Bruker D2 PHASER diffractometer equipped with a Cu $K_{\alpha 1}$(λ=1.54056 Å) and $K_{\alpha 2}$(λ=1.54439 Å) radiation source with the ratio of 2:1. Diffraction pattern was collected with the scan range (2θ) between 10° and 100°, a step size of 0.081°, and a counting time of 0.5 s per step. Membrane samples were measured in ambient conditions before electrolysis and again within 30 min after operation.

2. Flow cell experiments

The flow cell (Figure S1) was assembled with two compartments separated by a 99.99% pure Pd membrane, sealed with gaskets to prevent electrolyte crossover. All the experiments were carried out under ambient conditions. The aqueous compartment contained an IrO_2-coated platinized Ti mesh anode backed with a Pt back plate to prevent leakage. The non-aqueous compartment contained a stainless-steel mesh cathode serving as the gas diffusion electrode, positioned between the non-aqueous electrolyte chamber and the gas flow field. A delithiated lithium iron phosphate (LFP) electrode, clipped via a copper wire, served as the reference electrode in the non-aqueous compartment. A metal foil current collector was placed in contact with the membrane edge, avoiding the active area, to enable membrane potential measurements.
A Biologic SP-150 two-channel potentiostat was used. Before each experiment, a "pre-hydridation" step was performed by configuring the electrical circuit as shown in Figure S2 (pre-hydridation channel), with the Pd or Pt membrane connected as the cathode, the IrO_2-coated Ti mesh as the anode. Then, a total of 20 ml of 0.1 M $NaClO_4$ (prepared in either H_2O or D_2O) was supplied to the aqueous chamber at a flow rate of 3 ml min^{-1}. The electrolyte was stored in a reservoir bottle fitted with vent holes in the cap to allow O_2 generated in the aqueous chamber to escape. A constant current of -9 mA cm^{-2} was applied for 45 minutes.

After pre-hydridation, the cell was purged with N_2 gas for 30 minutes, electrolyte lines were cleaned by circulating dried diglyme for 10 minutes, and gas traps were prepared by filling 100 ml of water into the measuring cylinders. The N_2 gas trap contained 250 µl of 4 M HCl to capture NH_3 as NH_4Cl. The gas and electrolyte lines were then purged with N_2 and connected to the electrolyte prepared in a vial with a septum cap.

For the main N_2 reduction experiments, the aqueous electrolyte was kept flowing at 3 ml min^{-1}. The cell was reconfigured as shown in Figure S2 (N_2 reduction channel), with the cathode connected to the stainless-steel mesh, IrO_2-coated Ti mesh anode, and LFP reference electrode to drive the electrochemical reactions. The second potentiostat channel was connected to the Pd membrane and the reference electrode to monitor the membrane potential. During operation, N_2 was flowed at a rate of 30 ml min^{-1}, and 12.5 ml of the non-aqueous electrolyte (1M LiBF4 in diglyme with 0.3%EtOH) in a septum vial was flowed to the cell non-aqueous chamber at a rate of 3 ml min^{-1}. The triple-phase boundary was established by adjusting the height of the gas exhaust in the water column until a continuous flow of electrolyte was visible and flooding of the gas diffusion electrode was avoided.

2.1 54C prehydride+162C N_2 reduction flow cell experiments:

After 54C prehydride. Potentistatic electrochemical impedance spectroscopy (PEIS) was used to obtain the resistance between cathode and anode. Linear sweep voltammetry (LSV) was performed by scanning the cathode potential from 0 V to –4 V *vs.* a LFP reference at a scan rate of 20 mV s^{-1}, terminating when the current density reached 1.75 mA cm^{-2}_{geo}. Then, a pulsing current density of –6 mA cm^{-2}_{geo} was applied for 1min on +1min rest for 200 cycles until 162C charge had passed. Non-aqueous electrolyte was flowing from a septum vial to the cell at a flow rate of 3 ml min^{-1}. An aliquot of the non-aqueous electrolyte 200 μL was collected after 18C, 36C, 54C, 72C, 90C, 108C and 162C had passed for NH_3 quantification in ion chromatography.

2.2 10C N_2 reduction flow cell experiments:

After 54C prehydride. Potentistatic electrochemical impedance spectroscopy (PEIS) was used to obtain the resistance between cathode and anode. LSV was performed by scanning the cathode potential from 0 V to –4 V *vs.* a LFP reference at a scan rate of 20 mV s^{-1}, terminating when the current density reached 1.75 mA cm^{-2}_{geo}. Then, a constant current density of –1.75 mA cm^{-2}_{geo} was applied for 2500 s (-2.7 mA cm^{-2}_{geo} for 1667 s for EC-MS experiments), followed by a 30-minute open-circuit. Both NH_3 trapped in the electrolyte and in the acid trap at NH_4^+ were quantified using ion chromatography.

2.3 Blank experiment for water concentration:

The experiment was carried out by flowing 12.5 ml of the non-aqueous electrolyte (1M $LiBF_4$ in diglyme with 0.3% EtOH) and balancing with the inert gas N_2. A Pd/Pd black membrane was used. No aqueous electrolyte was flowed in the aqueous chamber. The electrolyte was sampled at 0 hours (before connecting to the cell), 1 hour, and 5 hours.

3. <u>Flow cell connected to an electrochemistry mass spectrometry experiment (EC-MS)</u>

The flow cell was connected to the Spectro inlet electrochemistry mass spectrometer (EC-MS) professional, equipped with a Quadrupole Mass Spectrometer (QMG 250 from PFEIFFER) (Figure S9). A spectro inlet aqueous-aqueous chip (non-aqueous chip should also be delicate) was used to allow a tiny fraction of gas flow from the non-aqueous compartment to diffuse into the mass spectrometer. The majority of the outlet gas was still bubbled into the acid trap. A

wpectro inlet aqueous cell was mounted on top of the chip to provide a gas-tight environment and allow gas tube connection.

EC-MS settings: Soft ionization energy of 26 eV, as reported by Fu et al., was used to suppress the water fragment at m/z=17 [5]. The Secondary Electron Multiplier (SEM) was set to 1500 V to allow maximum signal amplification. Signals for species of m/z=2 (H_2), m/z=3 (HD), m/z=4 (D_2), m/z=16 (CH_4), m/z=17 (NH_3), m/z=18 (H_2O or NDH_2), m/z=19 (ND_2H), m/z=20 (ND_3) and m/z=32 (O_2) were selected to be monitored during the experiments. The chip pressure was set to 0 mbar to allow the maximum amount of gas product to diffuse into the MS for detection (Figure S9).

After performing pre-hydridation and cell purging (see section above). The non-aqueous electrolyte began to flow at a rate of 3 ml/min. The triple-phase boundary was balanced by flowing N_2 at a rate of 30 ml min^{-1} (we were able to achieve negligible flooding by cutting a perfect gasket for the stainless-steel mesh; however, if any non-aqueous electrolyte touches the chip, the experiment would be terminated). Then, after fully balancing the cell, the N_2 flow rate was gradually reduced by 2 ml min-1 every 5 minutes until an N_2 flow rate of 5 ml min^{-1} was achieved. This step maximized the concentration of NH_3 in the N_2 outlet gas stream. Then, with SEM off, the cell was left for one hour to allow all the mass signals to stabilize. After that, SEM was turned on and the cell was left for another 30 minutes to allow the signal to decay further. Once the SEM was on, the observed decay could arise not only from changes in the surrounding environment but also from detector-related effects, such as dynode surface charging, contamination, or gain drift. Because the secondary electron multiplier gains decay relatively quickly with use, part of the signal drop may also result from the gradual loss of amplification efficiency over time [6].

Finally, after electrochemical measurements, both NH_3 trapped in the electrolyte and in the acid trap were quantified using ion chromatography to calculate the Faradaic efficiency.

Note: We were unable to perform MS measurements for more than 2 hours because tiny electrolyte droplets inevitably enter the gas-outlet line during the initial flow-balancing step. Although the droplets do not immediately reach the MS chip, they eventually travels downstream and triggers the instrument's safety shutdown. Consequently, we must terminate the experiment early, which limits the total NH_3 produced and further reduces the likelihood of detecting ND_3.

4. <u>Calculations</u>

4.1 Faradaic efficiency (FE)

NH_3 can be trapped in both a non-aqueous electrolyte and an acid trap as NH_4^+, therefore $Eq. S1$ is used to calculate FE towards NH_3:

$$FE_{NH_3}(\%) = \frac{z * F(C_1V_1 + C_2V_2)}{Q} * 1 (Eq. S1)$$

Where FE_{NH_3} is the Faradaic efficiency towards NH_3 (%); F is the Faraday constant=96485 (C mol^{-1}); n is the number of electrons required to produce one molecule of NH_3 (n = 3); C_1 (mol dm^{-3}) is the concentration of NH_3 trapped as NH_4^+ in non-aqueous electrolyte; C_2 (mol dm^{-3}) is the concentration of gas phase NH_3 trapped as NH_4^+ in the acid trap; V_1 and V_2 (dm^3) are the

corresponding volume of the non-aqueous electrolyte and the acid trap; Q (C) is the charge passed during the experiments.

4.2 Energy efficiency

Energy efficiency (EE) was calculated as the ratio of the Gibbs free energy stored in the produced ammonia ($\Delta G_{NH_3} = 339$ kJ mol^{-1}) to the total electrical energy supplied to the electrochemical system, defined by Lazouski et al. [7]. Therefore, Equations S2 to S5 were used to calculate the EE (%):

$$E_{prehydridation} = \int V_{cell-prehydridation} * I_{prehydridation}\, dt \; (Eq. S2)$$

Where $E_{prehydridation}$ (kJ) is the energy input during prehydridation = 0.165 (kJ); $V_{cell-prehydridation}$ (V) is the cell voltage recorded during prehydridation; $I_{prehydridation}$ (A) is the current recorded during the prehydridation; dt (s) is the corresponding time interval.

$$E_{N_2\,reduction} = \int V_{cell-N_2\,reduction} * I_{N_2\,reduction}\, dt \; (Eq. S3)$$

Where $E_{N_2\,reduction}$ (kJ) is the energy input during N_2 reduction = 0.066 (kJ); $V_{cell-N_2\,reduction}$ (V) is the cell voltage recorded during N_2 reduction; $I_{N_2\,reduction}$ (A) is the current recorded during the N_2 reduction; dt (s) is the corresponding time interval.

$$n_{NH_3} = C_1V_1 + C_2V_2 \; (Eq. S4)$$

Where n_{NH_3} (mol) is the amount of NH_3 produced during N_2 reduction.

$$EE\,(\%) = \frac{n_{NH_3} * \Delta G_{NH_3}}{E_{prehydridation} + E_{N_2\,reduction}} * \; (Eq. S5)$$

4.3 Average current density

The average current density ($J_{average}$) was calculated as the total charge passed (Q) during the N_2 reduction experiment divided by the electrolysis time (t) and the electrode surface area (A). Therefore, Equation S6 was used:

$$J_{average} = \frac{Q}{A * t} (Eq. S6)$$

4.4 Effect of including pre-hydridation charge of 54 C during Faradaic efficiency (FE) calculation

If we are calculating the FE includes the prehydridation charge, we can then write the equation as:

$$FE_{(with\,prehydridation)} = \frac{n_{NH_3}(t) * z * F}{Q_{N_2\,reduction}(t) + 54} (Eq. S7)$$

Where $n_{NH_3}(t)$ is the amount of NH_3 produced at time t; z is the number of electrons required to form one NH_3 molecule; F is the Faraday constant.

As more charge is passed, $Q_{N_2\,reduction}(t) \gg 54$, we can therefore write the equation as:

$$FE_{(with\,prehydridation)} = \frac{n_{NH_3}(t) * z * F}{Q_{N_2\,reduction}(t)} Eq. S8)$$

This indicates that the influence of the 54 C pre-hydridation charge diminishes with increasing reaction time and would eventually become negligible, assuming we are constantly generating NH_3. As shown in Figure S6, Faradaic efficiency appears artificially low at early stages because the fixed pre-hydridation charge dominates the denominator of the Faradaic efficiency calculation. Consequently, the Faradaic efficiency increases and approaches a steady-state value that reflects the true Faradaic efficiency of the N_2 reduction process. However, I believe the charge amount (162C) we passed for N_2 reduction is not sufficient for Faradaic efficiency to reach a steady state yet as the Faradaic efficiency without pre-hydridation charge remains stable at around 50% (See Table 2). We predict that if more charge is passed the Faradaic efficiency (with pre-hydridation) will eventually reach 50%.

5. Figures

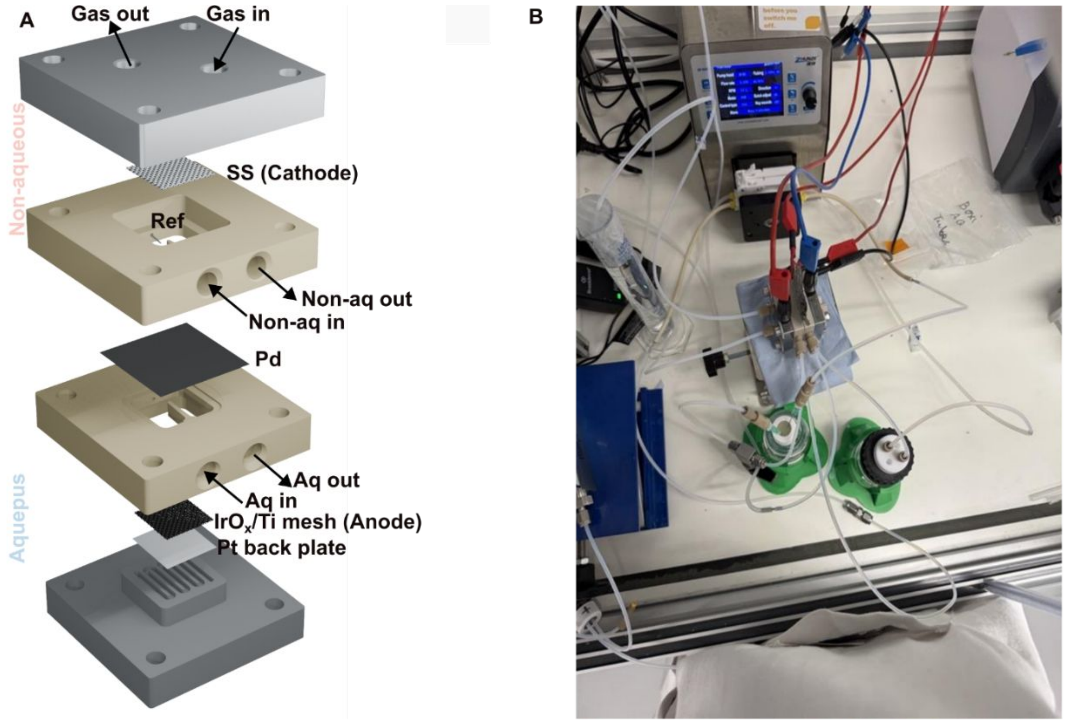

Figure S1. Image of the two-compartment flow cell used for electrochemical measurement. 2.25 cm² active electrode area, with a 4 mm separation between the cathode and anode. (A) 3D pictures of separate parts of the flow cell. With two stainless steel gas flow fields and two PEEK electrolyte flow fields (no gas was flowed in the aqueous compartment). The distance between the Anode and the membrane is 3.42 mm. The distance between the cathode and the membrane is 3.42 mm. Both the aqueous and non-aqueous electrolyte chambers have a volume of 1 ml. (B) Picture of an assembled cell for the experiment.

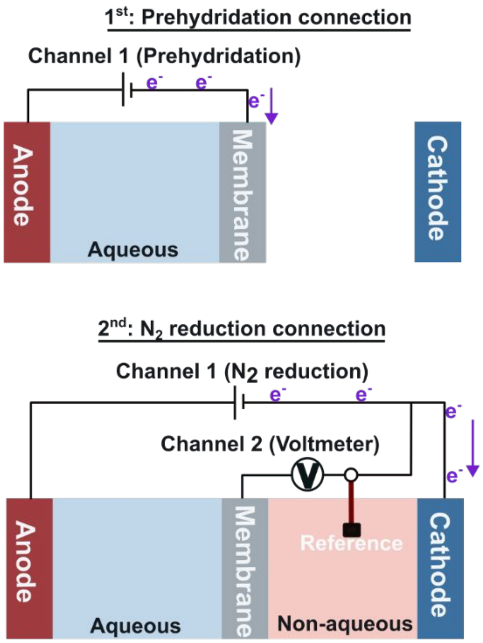

Figure S2. Electric circuit connection. Prehydridation connection was first performed by connecting a Pd membrane as a cathode. Secondly, a two-channel potential stat was synchronized, Channel 1 connects to the anode, cathode, and reference electrodes. Channel 2 connects to the membrane and the reference electrode (Note: only reference cables were used for Channel 2; power cables were disconnected to avoid current leakage).

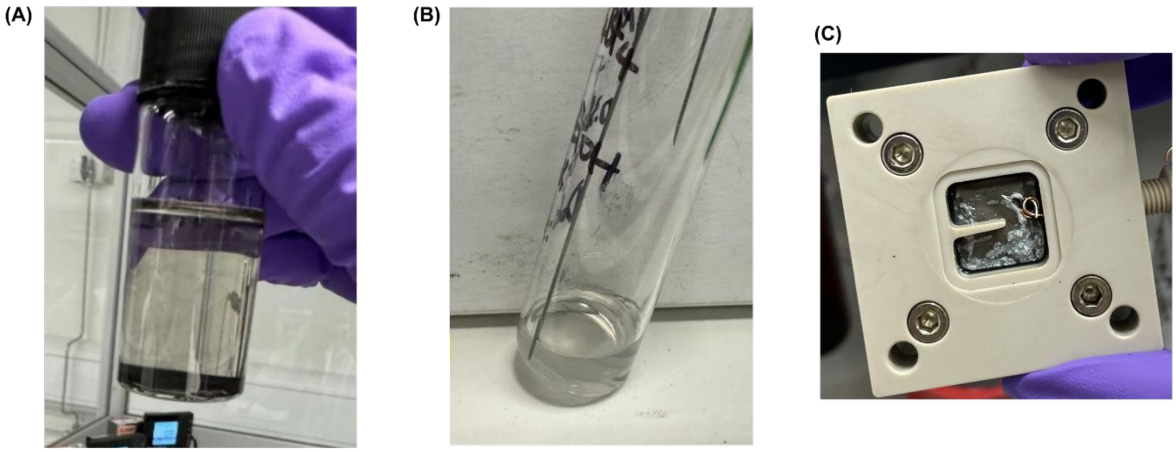

Figure S3. (A) Black precipitation that is formed without the prehydride step on the Pd membrane. (B) cloudy electrolyte after 162C was passed for the prehydrided Pd/Pd black. (C) The thick solid-electrolyte interface (SEI) formed after 162C was passed with using the optimized pulsing strategy.

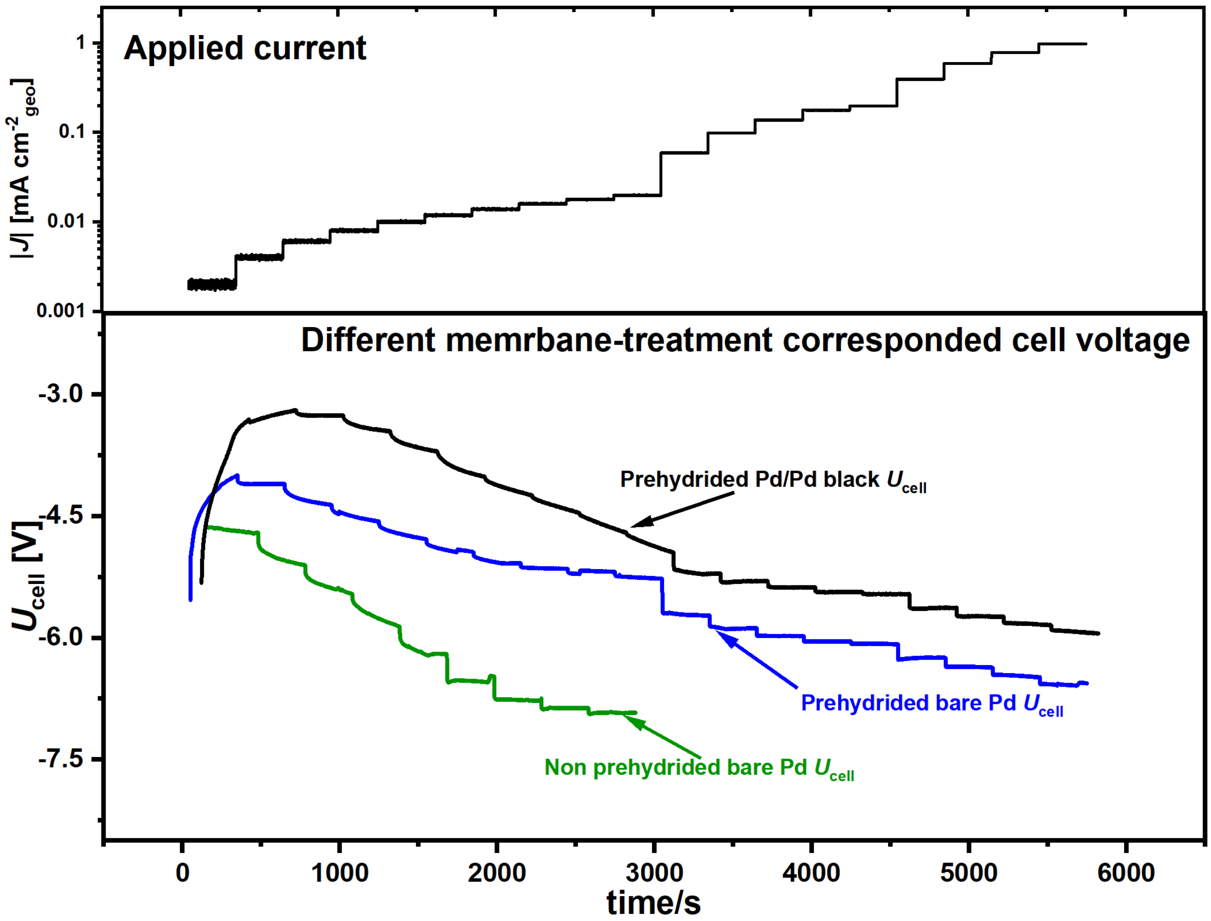

Figure S4. Cell voltage was measured at different constant current steps when using Pd membranes with various types of pre-treatments. Non-prehydride bare Pd (Green), introducing a cell voltage of more than 1V, compared with Prehydrided bare Pd (Blue) and Prehydrided Pd/Pd black (Black).

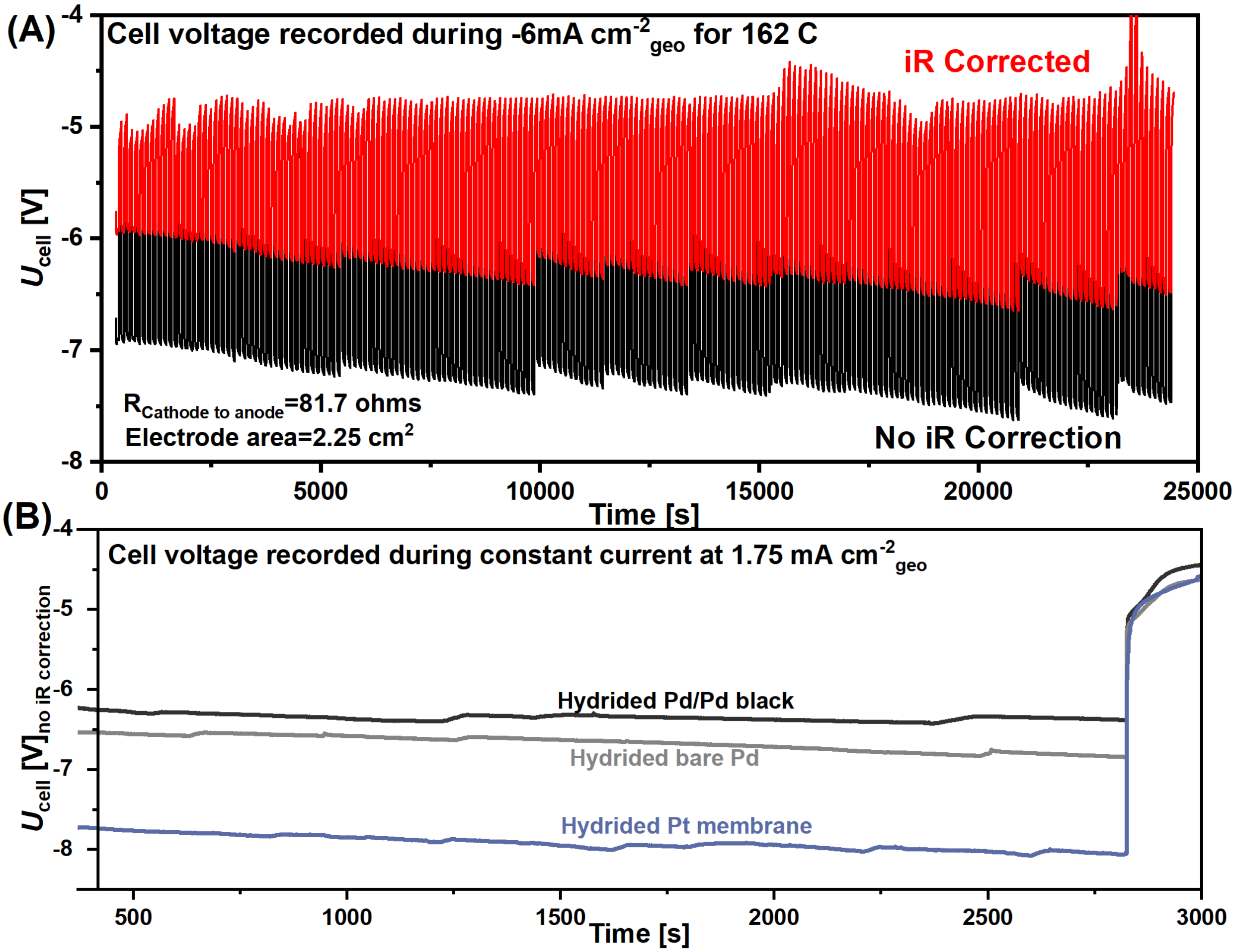

Figure S5. (A) Cell voltage recorded during pulsing current at -6 mA cm^{-2}_{geo} for 162 C with (red) and without (black) iR correction. The Resistance between cathode and the anode ($R_{cathode\ to\ anode}$) obtained from potentistatic electrochemical impedance spectroscopy (PEIS) (B) Cell voltage recorded during constant current at 1.75 mA cm^{-2}_{geo}, when pre-hydrided Pd/Pd black, Pd and Pt were used.

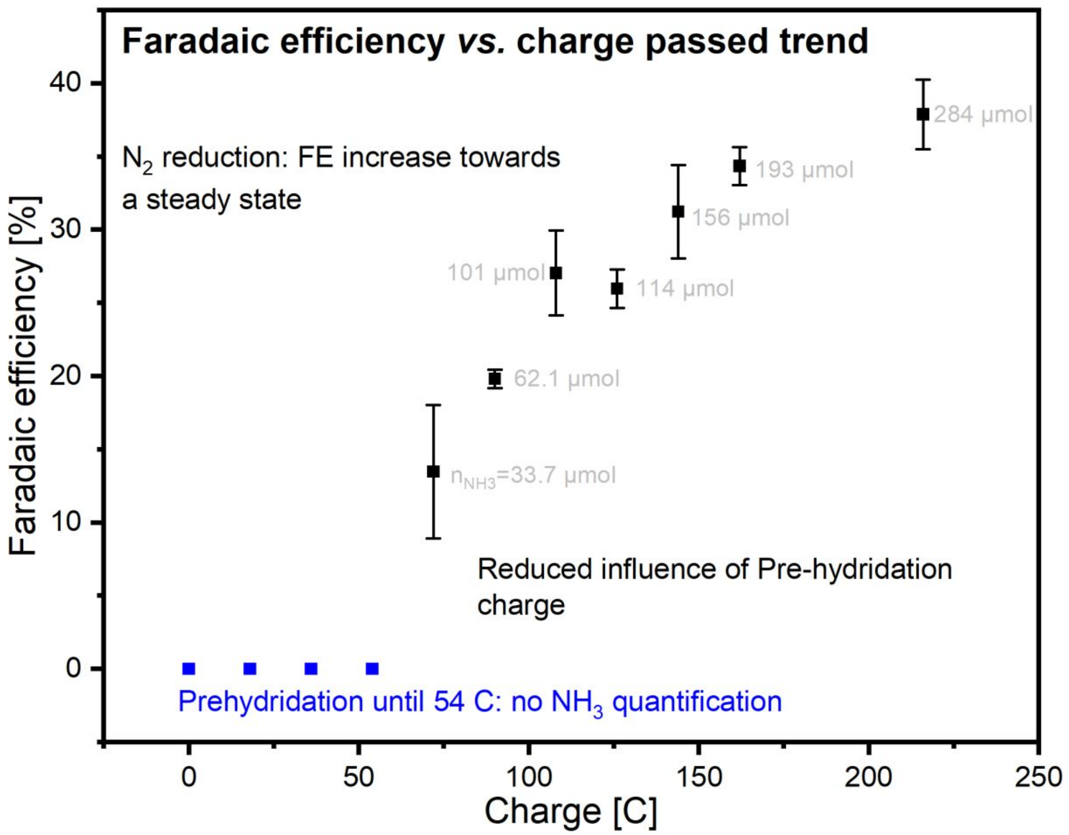

Figure S6. Trend of Faradaic efficiency calculated with the 54 C pre-hydridation charge included. No NH_3 was detected during the pre-hydridation stage (0–54 C). As N_2 reduction proceeds (a further 162 C), the fixed 54 C contributes less to the total charge, resulting in an increasing Faradaic efficiency that approaches its intrinsic value.

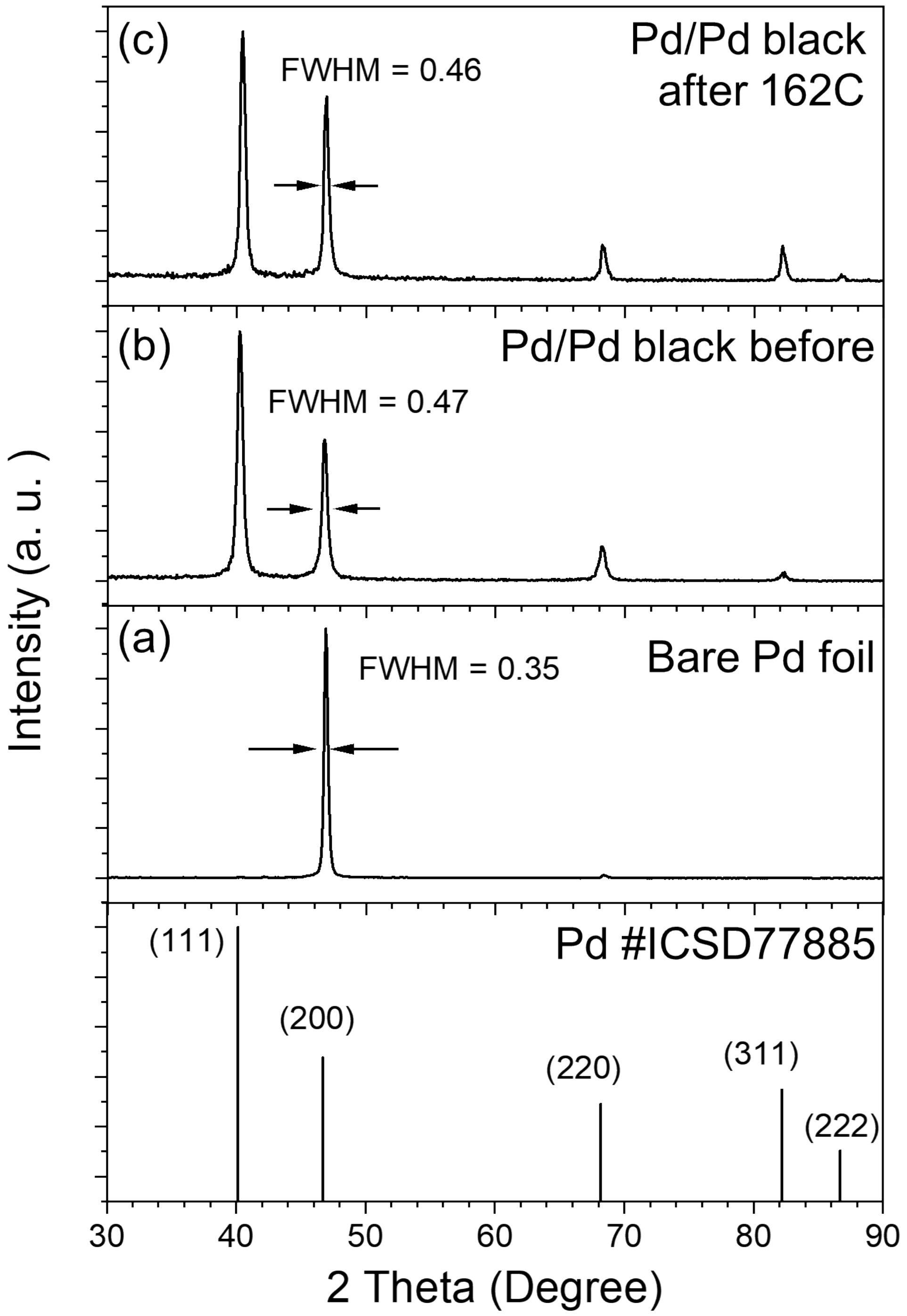

Figure S7. X-ray diffraction (XRD) patterns of the Pd membrane at different stages of preparation and operation.(a) XRD patterns of the pristine Pd foil, showing a preferred orientation of the (100) facet. (b) XRD patterns of the electrodeposited Pd black on the foil surface. The significant peak broadening indicated by the full width at half maximum (FWHM) of the Pd (200) was observed, presumably caused by the smaller crystallite size of a nanostructured Pd-black compared with the pristine Pd foil. (c) XRD pattern of Pd black after passing 162 C of charge under the N2 reduction conditions, measured within 30 min of

completing the electrolysis. Negligible changes in the peak position and width were observed relative to the Pd black sample before N_2 reduction, presumably due to the limited resolution of the benchtop diffractometer. Further synchrotron experiments to characterize the Pd membrane will be conducted.

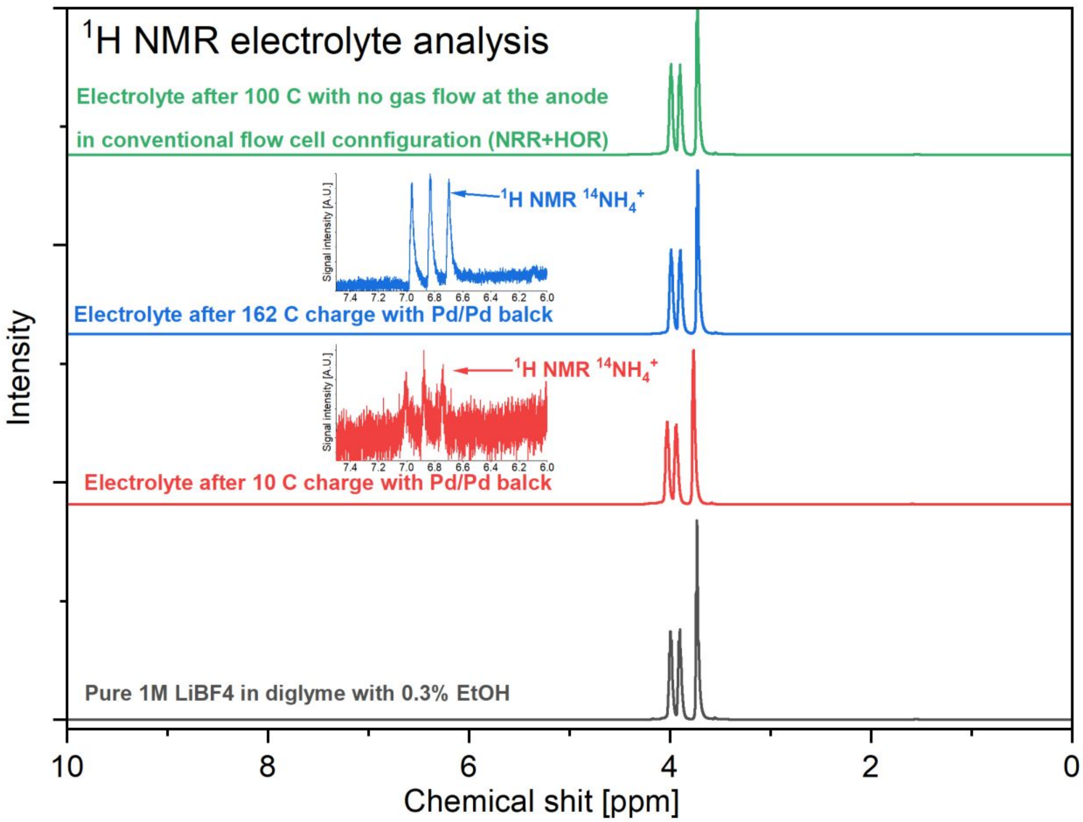

Figure S8. ^{1}H NMR spectra of the fresh electrolyte (1 M $LiBF_4$ in diglyme with 0.3% EtOH) compared with the electrolyte after experiments using different membranes and charge passed. The spectra show characteristic signals of diglyme (δ ≈ 3.1–3.9 ppm). No deuterated lock solvent was added for these measurements. Due to overlapping signals from diglyme and ethanol (δ≈ 3.6 ppm), it is challenging to deconvolute and quantify the proton-donor concentration over time in this specific solvent system. The triplet peaks at δ ≈ 6.0–7.0 ppm indicate the presence of Ammonium.[8]

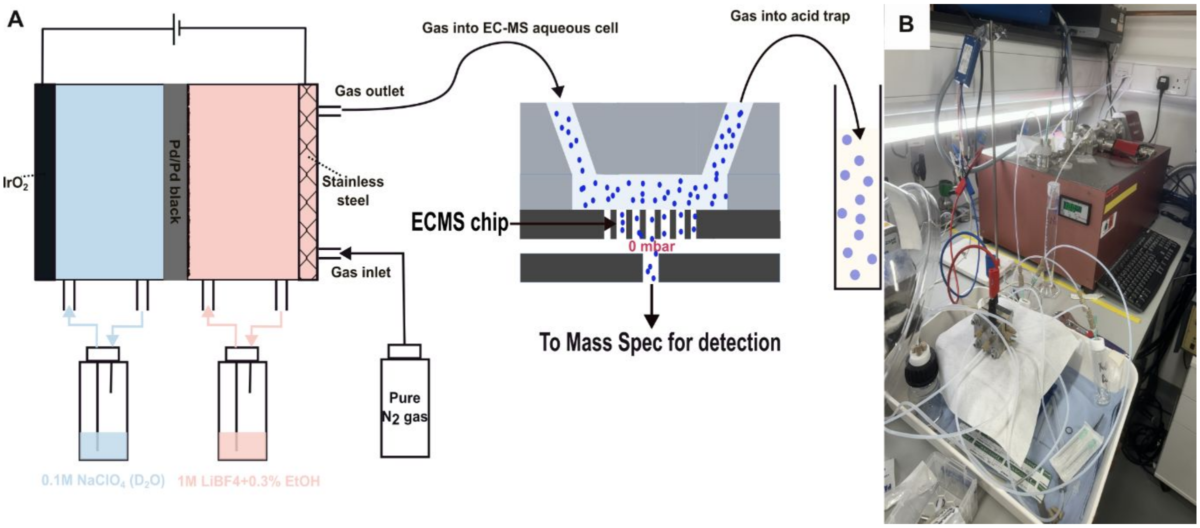

Figure S9. Configuration of connecting the flow cell to the EC-MS. (A) 2D plot demonstrates the flow path of gas. (B) Photograph of the experimental setup.

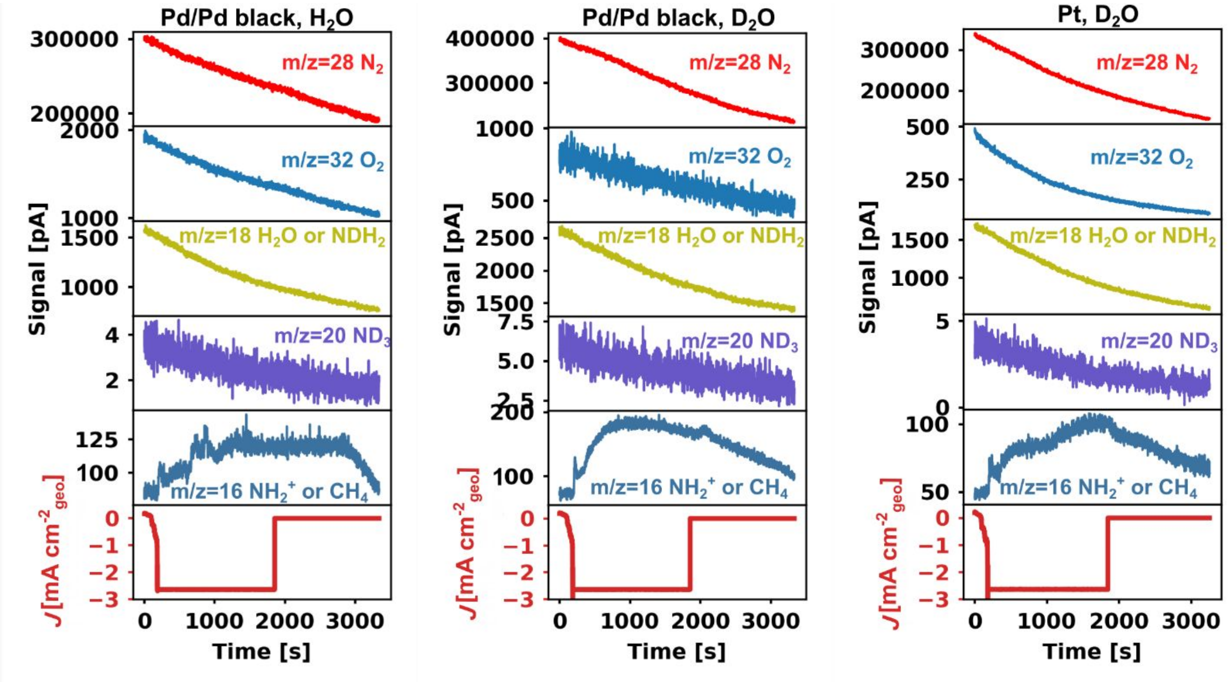

Figure S10. Additional MS signals were recorded when using H_2O and D_2O as proton sources. m/z=28 (N_2) showed a signal decay even though the flow rate was held constant at 5 ml min^{-1} due to the use of SEM; m/z=32 (O_2) signals were monitored to ensure no ingress of air occurred from the surroundings; m/z=18 (H_2O or NDH_2) signals were observed (Failed to detect NDH_2 due to constantly dropping H_2O signal, maybe require EC-MS vacuum chamber heating to lower the H_2O baseline and stay constant); m/z=20 (ND_3) signals were monitored but failed due to insufficient H/D exchange (see the main text); m/z=16 (NH_2^+ or CH_4) signals started to increase as soon as the voltage to the cell was applied indicating CH_4 formation from ethanol reduction [9], the delayed signal dropping may due to NH_2^+ as a fragment of NH_3, where NH_3 signal was also involved.

6. Tables

Dilution factor	Water content (ppm)	Undiluted water content (ppm)
1600 times	323	516800
800 times	542	433600
400 times	833	333200
		Average = 427900

Table 1. Karl fisher titration for Nafion membrane example calculation table.

Membrane	Current density (mA cm^{-2}) until 10 C passed	Charge passed (C)	Amount of ammonia produced (µmol)	Faradaic efficiency (Pre-hydridation charge included) (%)	Faradaic efficiency (No pre-hydridation charge) (%)	Water concentration (ppm)
Pd/Pd black	-6	162	271	36.4	48.5	335
Pd/Pd black	-6	162	297	40.0	53.0	269
Pd/Pd black	-6	162	244	32.7	43.6	284
Pd/Pd black (EC-MS, H_2O)	-2.70	10	28.0	12.5	80.0	357
Pd/Pd black (EC-MS, D_2O)	-2.70	10	28.0	12.5	80.0	150
Pt membrane (EC-MS, D_2O)	-2.70	10	24.0	11.0	70.5	328
Pd/Pd black	-1.75	10	28.4	12.9	82.3	116
Pd/Pd black	-1.75	10	20.1	10.7	60.0	120
Pd/Pd black	-1.75	10	24.6	11.1	71.2	142
Bare Pd	-1.75	10	27.1	12.3	78.3	133
Bare Pd	-1.75	10	24.2	11.0	70.0	162
Pt	-1.75	10	30.0	13.6	86.0	118
Nafion	-1.75	10	1.50	0.68	4.34	427900
Nafion	-1.75	10	1.84	0.83	5.32	630000
Pd/Pd black (blank experiment for water concentration)	none	none	none	none	none	0 hours: 148<br>1 hour: 160<br>5 hours: 270

Table 2. Summary of the experiments carried out for various membranes and conditions.

Charge passed (C)	Cumulative NH_3 yield (μmol)		Faradaic efficiency (with pre-hydridation, 54 C)	Faradaic efficiency (no pre-hydridation charge)
	Average	Standard deviation		
18	36.4	9.30	13.5	54.2
36	69.6	13.1	19.8	50.0
54	100	7.90	27.0	54.1
72	119	10.2	26.0	45.8
90	151	14.4	31.2	50.1
108	189	8.40	34.3	51.7
162	271	26.2	37.9	50.7

Table 3. Summary of experiments carried out with a Pd/ Pd black membrane at -6 mA cm^{-2} until 162 C was passed with an aliquot from the electrolyte collected after 18, 54, 72, 90, 108 and 162 C had passed from n= 3 independent experiments

References

(1) Fink, A. G.; Delima, R. S.; Rousseau, A. R.; Hunt, C.; LeSage, N. E.; Huang, A.; Stolar, M.; Berlinguette, C. P. Indirect H2O2 synthesis without H2. *Nature Communications* **2024**, *15* (1), 766.

(2) Mohsen Ismail, A.; Kortlever, R. Morphology-Controlled Electrodeposition of Copper Gas Diffusion Electrodes for CO2 Electroreduction. *ACS Applied Energy Materials* **2025**, *8* (12), 8551-8560. DOI: 10.1021/acsaem.5c01142.

(3) Tort, R.; Westhead, O.; Spry, M.; Davies, B. J. V.; Ryan, M. P.; Titirici, M.-M.; Stephens, I. E. L. Nonaqueous Li-Mediated Nitrogen Reduction: Taking Control of Potentials. *ACS Energy Letters* **2023**, *8* (2), 1003-1009. DOI: 10.1021/acsenergylett.2c02697.

(4) Burdis, C.; Tort, R.; Winiwarter, A.; Rietbrock, J.; Barrio, J.; Titirici, M. M.; Stephens, I. E. A carbon cathode for lithium mediated electrochemical ammonia synthesis. *Energy & Environmental Science* **2025**.

(5) Fu, X.; Pedersen, J. B.; Zhou, Y.; Saccoccio, M.; Li, S.; Sažinas, R.; Li, K.; Andersen, S. Z.; Xu, A.; Deissler, N. H. Continuous-flow electrosynthesis of ammonia by nitrogen reduction and hydrogen oxidation. *Science* **2023**, *379* (6633), 707-712.

(6) Yilmaz, T. Investigation of Electrocatalytic Oxidation Reactions: Insights From Electrochemical Mass Spectrometry. **2023**.

(7) Lazouski, N.; Schiffer, Z. J.; Williams, K.; Manthiram, K. Understanding continuous lithium-mediated electrochemical nitrogen reduction. *Joule* **2019**, *3* (4), 1127-1139.

(8) Bemana, H.; Schumann, H.; McKee, M.; Nozinovic, S.; Daniels, J.; Weisbarth, R.; Kornienko, N. Accelerating lithium-mediated nitrogen reduction through an integrated palladium membrane hydrogenation reactor. *Nature Communications* **2025**, *16* (1), 6696. DOI: 10.1038/s41467-025-62088-z.

(9) Krempl, K.; Hochfilzer, D.; Cavalca, F.; Saccoccio, M.; Kibsgaard, J.; Vesborg, P. C.; Chorkendorff, I. Quantitative operando detection of electro synthesized ammonia using mass spectrometry. *ChemElectroChem* **2022**, *9* (6), e202101713.
